# A σ^E^-Mediated Temperature Gauge Controls a Switch from LuxR-Mediated Virulence Gene Expression to Thermal Stress Adaptation in *Vibrio alginolyticus*

**DOI:** 10.1371/journal.ppat.1005645

**Published:** 2016-06-02

**Authors:** Dan Gu, Min Guo, Minjun Yang, Yuanxing Zhang, Xiaohui Zhou, Qiyao Wang

**Affiliations:** 1 State Key Laboratory of Bioreactor Engineering, East China University of Science and Technology, Shanghai, China; 2 Shanghai—MOST Key Laboratory of Health and Disease Genomics, Chinese National Human Genome Center at Shanghai, Shanghai, China; 3 Shanghai Collaborative Innovation Center for Biomanufacturing Technology, Shanghai, China; 4 Shanghai Engineering Research Center of Maricultured Animal Vaccines, Shanghai, China; 5 Department of Pathobiology and Veterinary Science, University of Connecticut, Storrs, Connecticut, United States of America; University of California Davis School of Medicine, UNITED STATES

## Abstract

In vibrios, the expression of virulence factors is often controlled by LuxR, the master quorum-sensing regulator. Here, we investigate the interplay between LuxR and σ^E^, an alternative sigma factor, during the control of virulence-related gene expression and adaptations to temperature elevations in the zoonotic pathogen *Vibrio alginolyticus*. An *rpoE* null *V*. *alginolyticus* mutant was unable to adapt to various stresses and was survival-deficient in fish. In wild type *V*. *alginolyticus*, the expression of LuxR-regulated virulence factors increased as the temperature was increased from 22°C to 37°C, but mutants lacking σ^E^ did not respond to temperature, indicating that σ^E^ is critical for the temperature-dependent upregulation of virulence genes. Further analyses revealed that σ^E^ binds directly to -10 and -35 elements in the *luxR* promoter that drive its transcription. ChIP assays showed that σ^E^ binds to the promoter regions of *luxR*, *rpoH* and *rpoE* at high temperatures (e.g., 30°C and 37°C). However, at higher temperatures (42°C) that induce thermal stress, σ^E^ binding to the *luxR* promoter decreased, while its binding to the *rpoH* and *rpoE* promoters was unchanged. Thus, the temperature-dependent binding of σ^E^ to distinct promoters appears to underlie a σ^E^-controlled switch between the expression of virulence genes and adaptation to thermal stress. This study illustrates how a conserved temperature response mechanism integrates into quorum-sensing circuits to regulate both virulence and stress adaptation.

## Author Summary

Zoonotic *Vibrio* outbreaks are believed to be closely associated with increases in environmental temperature. The mechanisms underlying this phenomenon have not been defined. Here, we show that the expression of the *V*. *alginolyticus* exotoxin Asp and other quorum-sensing (QS)-regulated virulence factors are induced by increasing temperatures, with the maximum expression observed at approximately 37°C. σ^E^ plays an essential role in regulating the QS master regulator LuxR in response to temperature shifts by binding directly to the -10 and -35 regions of the *luxR* promoter to drive its transcription. However, at higher thermal stress temperatures, σ^E^ binding to the *luxR* promoter decreased, resulting in a reduction in *luxR* transcription. This change underlies a binomial switch mechanism that regulates σ^E^-controlled virulence gene expression patterns. Furthermore, we found that anti-σ^E^ signaling was involved in this stress and virulence reciprocal switch. This study suggests that a common temperature response mechanism is integrated into QS circuits to regulate both virulence and adaptation in related *Vibrio* taxa.

## Introduction

It has been established that warming sea surface temperatures (SST) threatens marine ecosystems, including coral species that reside in coral reefs [1,2]. In addition, elevated sea temperatures are associated with mass mortalities in farmed sea animals [3], and these are often associated with infection by vibrios [4,5]. Elevated SST increases the abundance of marine vibrios, including *V*. *coralliilyticus* and *V*. *cholerae* [6,7]. However, while temperature does appear to play a more direct and distinct role in temperature-related infection scenarios involving opportunistic vibrios [8–11], the mechanisms underlying this process are largely unknown. Furthermore, vibrios have to adapt to different temperatures, e.g., in the natural residence, during infection or at the extreme ocean conditions. The molecular mechanisms to coordinate gene expression in order to adapt to different temperatures have not been precisely elucidated in vibrios.

Alternative sigma (σ) factors are global regulators that enable bacterial pathogens to coordinate the expression of genetic traits associated with stress adaptation and virulence in response to diverse stimuli in both the environment and, more specifically, the host. By selectively using alternative sigma factors, bacterial pathogens can integrate stress adaptation pathways with virulence regulation circuits. Sigma factor E (σ^E^ or σ^24^), which is encoded by *rpoE*, is a key regulator of the extracytoplasmic stress response, and it plays essential roles in the pathogenesis of numerous bacterial pathogens, including various vibrios [12–16]. In *Escherichia coli*, *rpoE* is a component of a four-gene operon that also includes the genes encoding anti-sigma factor RseA, the negative regulator RseB and a positive modulator, RseC [17]. RseA, a single transmembrane protein that contains an N-terminal cytoplasmic domain and C-terminal periplasmic domain, tethers σ^E^ to its N-terminal domain and inhibits σ^E^ activity by blocking its association with RNA polymerase [17,18]. RseB, a periplasmic protein, binds to RseA’s periplasmic domain, thereby increasing the affinity of RseA for σ^E^ and consequently increasing the stability of RseA. RseC is an inner-membrane protein that can positively regulate σ^E^ activity via an unknown mechanism. Under stress conditions, RseA is cleaved by the transmembrane protease DegS following a regulated intramembrane proteolysis (RIP) cascade that releases σ^E^ [17]. Subsequently, the activated σ^E^ can incorporate the RNA polymerase core enzyme and trigger the transcription of specific sets of genes that are responsible for stress adaptations and virulence. In *E*. *coli*, the σ^E^ regulon consists of functionally conserved coherent envelope homeostasis-related genes and a variable portion of pathogenesis-associated organism-specific genes [19]. Remarkably, the genes for several sigma factors, including RpoE, RpoH, and RpoN, are part of the conserved σ^E^ regulon in *E*. *coli* [20], which reveals the presence of an “extended” regulatory strategy and a transcriptional circuitry to maintain the σ^E^ response [19].

Quorum-sensing (QS) is a bacterial cell-to-cell signaling system that regulates the expression of large numbers of genes in a cell density-dependent manner, often promoting bacterial virulence and stress responses [21–23]. In *V*. *harveyi*, three types of QS signaling molecules (i.e., autoinducers, AIs) are produced, including acylated homoserine lactones (HAI-1), furanosyl borate diester (AI-2), and CAI-1. The membrane sensors LuxN, LuxQ, and CqsS can detect their cognate AIs and transduce the signals required to trigger gene expression through phospho-relay cascades [24]. At low cell densities (low AI concentration), phosphates are directed by the kinases in upstream QS cascades to the signal relayer LuxU and pivotal regulator LuxO, which leads to the repression of master QS regulator (MQSR) LuxR expression by multiple *qrr* sRNAs (*qrr1*–*qrr5*) [24]. In contrast, at high cell density (high AI concentration), the phosphate circuit runs in the opposite direction, resulting in the dephosphorylation and inactivation of LuxO, which de-represses the expression of LuxR [24]. MQSR then binds to and regulates the expression of many (~150) target genes, leading to diverse phenotypes, including bioluminescence, biofilm formation, and motility [4,25]. Vibrios also deploy other regulators, such as AphA [26–28] and various *qrr-*mediated regulatory mechanisms to optimize the output of QS regulation. For the latter, it was recently discovered that *qrr3* uses four distinct mechanisms to regulate its cognate targets, demonstrating the presence of an exquisite and sophisticated regulatory architecture in the *V*. *harveyi* QS system [29]. Nevertheless, relatively little is known about how the genes encoding LuxR and other MQSR homologs are transcriptionally controlled by signals other than QS.

*V*. *alginolyticus* belongs to the *Harveyi* clade and is a normal inhabitant of coastal and estuarine environments in warm tropical regions and also represents one of the leading opportunistic pathogens in sea animals, including grouper, large yellow croaker, sea bream, Kuruma prawn, abalone, and carpet shell clam. This organism represents a sustained threat to aquaculture industries and ecosystems around the world [4]. Recently, *V*. *alginolyticus* was also identified as the etiological agent in coral bleaching, such as *Porites andrewsi* White syndrome (PAWS) [30]. In addition to sea animals, *V*. *alginolyticus* also causes superficial wound infections and other intra-/extra-intestinal diseases in humans [31,32]. *V*. *alginolyticus* pathogenicity is mainly associated with several extracellular proteases (ECPs), including two kinds of alkaline serine proteases, Asp and Pep, that act as exotoxins [33–37]. These factors are closely regulated by the *V*. *harveyi*-like QS system in *V*. *alginolyticus*, in which LuxO and LuxR play pivotal regulatory roles [36–42].

In this study, we explored the temperature dependence of the expression of Asp and other exotoxins. We found that the maximum level of Asp expression is reached at approximately 37°C and that its expression shuts down at 42°C, a temperature that triggers thermal stress in *V*. *alginolyticus*. In the nature, vibrios may adapt to such extreme marine environments, e.g., hot springs and hydrothermal vents that may reach 42°C or even higher. A mechanistic analysis revealed that σ^E^ mediates the temperature-dependent expression of *V*. *alginolyticus* exotoxins by controlling the expression of the MQSR LuxR. The σ^E^ signaling pathway is critical to switching from LuxR-dependent virulence gene expression at favorable temperatures to stress response-dependent gene expression at higher temperatures. Collectively, our data reveal a novel and sophisticated system of interplay between temperature-dependent σ^E^ signaling and QS circuits that control virulence and stress adaptation, and we suggest that may be common among *Vibrio* pathogens.

## Results

### Exotoxin expression is regulated by temperature in *V*. *alginolyticus*

**Fig 1:**
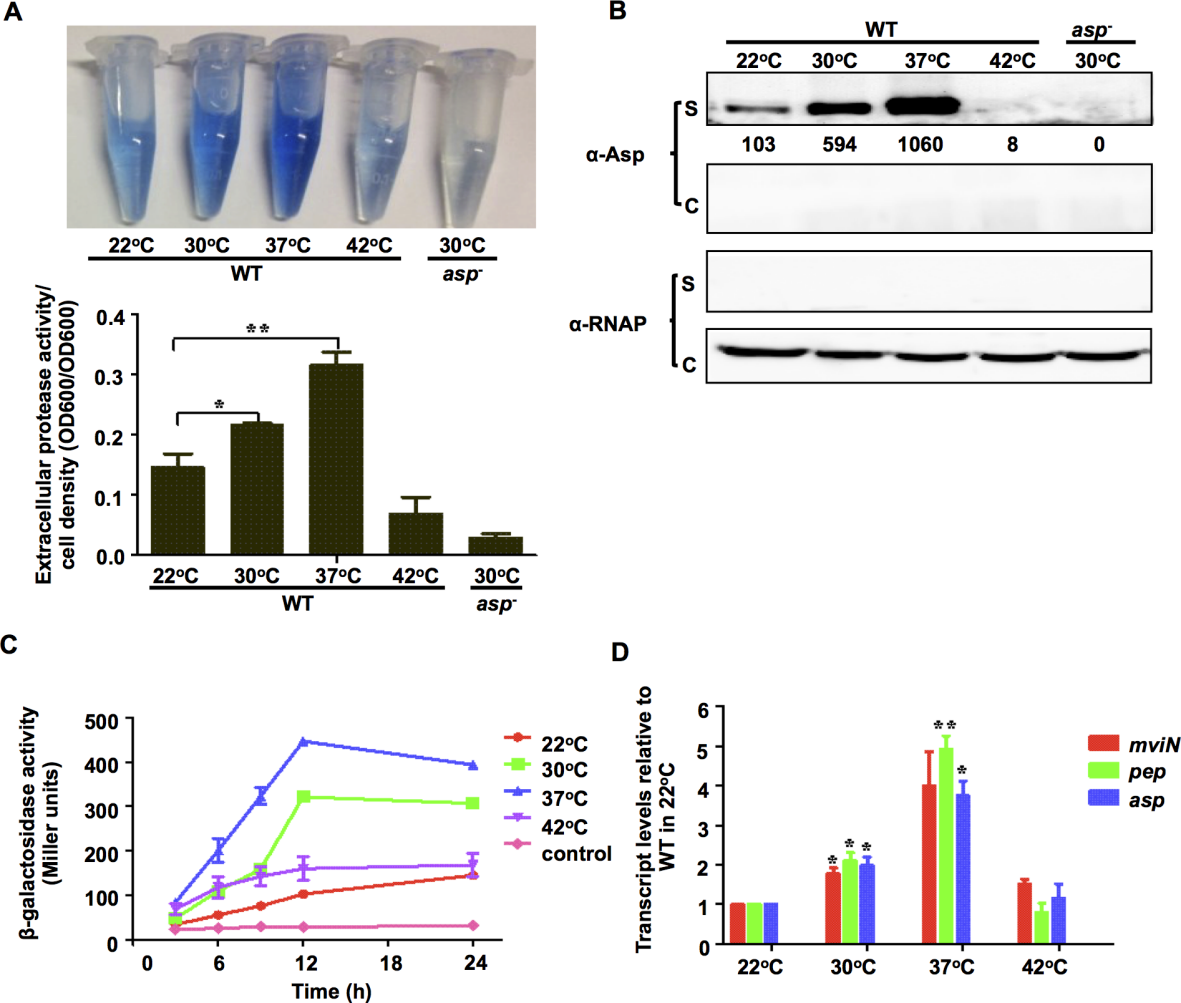
The expression of Asp and other extracellular toxins is controlled in a temperature-dependent manner. HPA digestion assays were performed to analyze extracellular Asp activity (**A**), and Western blot analysis was performed to determine Asp yields (**B**) in the wt strain at various temperatures. Bacterial cells were centrifuged after they were cultured in LBS medium for 9 h, and the supernatants were harvested to measure protease activity. Asp activity was normalized by dividing the total protease activity by the cell density for each strain. Western blot analysis was performed using concentrated supernatants (S) and cellular pellets (C) with Asp-specific antibodies. RNAP was used as the loading control for the blots. The numbers correspond to densitometry measurements. The results are presented as the mean ± S.D. (*n* = 3). *, *P*<0.05, and **, *P*<0.01, based on ANOVA comparisons. (**C**) P_*asp*_ *-lacZ* transcriptional analysis at different temperatures. The wt strain carrying the P_*asp*_-*lacZ* reporter plasmid was cultured in LBS and assayed to determine *β*-galactosidase activity. The results are shown as the mean ± S.D. (*n* = 3). (**D**) qRT-PCR analysis of the transcription of Asp and other virulence proteins (MviN and Pep) that were cultivated at different temperatures. Total RNA was isolated from the wt strain after it was grown in LBS at different temperatures for 9 h. qRT-PCR was performed as described in the Materials and Methods section. The results are shown normalized to the results for the 16S rRNA gene and were determined using the ΔΔ*C*_T_ method. The differences are shown relative to the levels in the wt cultures grown at 22°C. * *P* <0.05, *t-*test.

In these experiments, we investigated the mechanisms by which temperature modulates the production of the *V*. *alginolyticus* exotoxin Asp. *V*. *alginolyticus* grows fastest at temperatures ranging from 22°C to 37°C and slower at temperatures lower than 22°C or above 42°C. There was no apparent difference in growth rates in strains grown at temperatures ranging from 22°C to 37°C (S1A Fig) [43]. We analyzed the production of Asp in specimens grown at 22°C, 30°C, 37°C and 42°C, which reflects the SSTs in tropical regions (22–30°C) [44], the temperature of human hosts (37°C) during infection, as well as the temperature inducing thermal stresses (S1B Fig) in natural extreme environments (42°C). Asp activity increased as culture temperatures increased from 22°C to 37°C, but when the temperature reached 42°C, only minimal Asp activity was detected (Fig 1A). Asp activity is correlated with its secretion into the supernatant (Fig 1B). When Asp promoter activity was measured using *β*-galactosidase activities, it also increased as the temperature increased from 22°C to 37°C and dropped dramatically when the temperature reached 42°C (Fig 1C). Maximum P_*asp*_-*lacZ* activity was reached after the cells were cultured for 12 h at 30°C and 37°C, at which time the cells reached a stationary phase (S1A Fig). These results suggest that *asp* expression might be correlated with quorum-sensing (QS) [36,38] or the stationary phase sigma factor RpoS. Quantitative RT-PCR (qRT-PCR) analysis showed that cells grown at 37°C expressed 3.6-fold higher levels of *asp* transcripts than those grown at 22°C (*P*<0.05) (Fig 1D), indicating that temperature regulates *asp* expression at least in part at the transcriptional level. Similar results were observed for MviN and Pep (Fig 1D), two other exotoxins that are essential for pathogenesis [37,39,45]. Taken together, these data demonstrate that the transcription of Asp and other toxins (e.g., MviN and Pep) is temperature- dependent in *V*. *alginolyticus*.

### RpoE is essential for the temperature-dependent expression of Asp and other virulence factors

**Fig 2:**
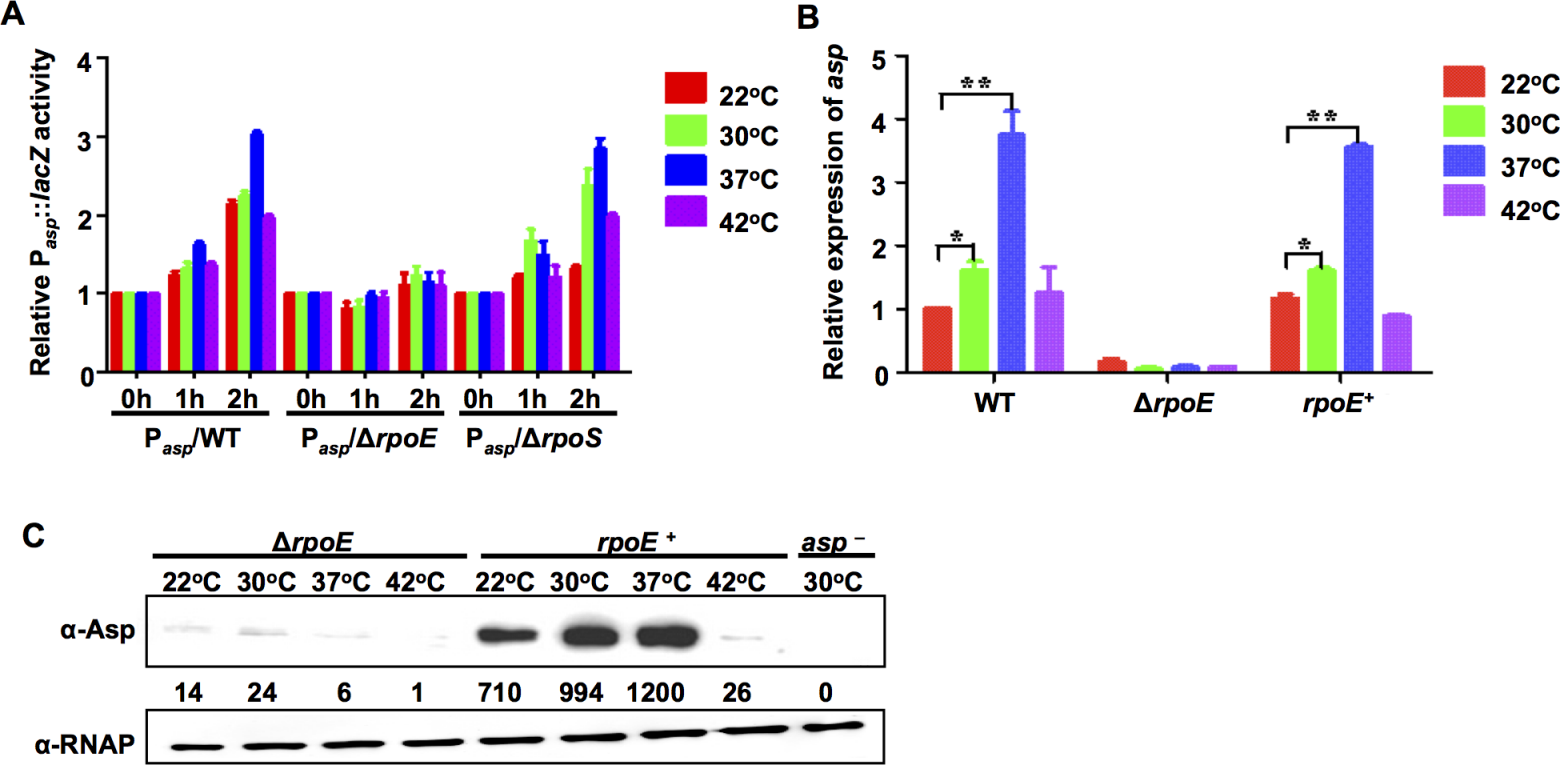
The expression of Asp is controlled by temperature via an RpoE-mediated mechanism. (**A**) The activity of P_*asp*_-*lacZ* in wt, Δ*rpoE*, and Δ*rpoS* strains. The strains were grown at 30°C to the exponential phase before the bacteria were shifted to various temperatures for 0, 1, and 2 h. Then, *β*-galactosidase activity was assayed. The results show the differences relative to the level of the same strain at 0 h. The results are shown as the mean ± S.D. (*n* = 3). (**B**) qRT-PCR analysis of *asp* transcription levels in wt, Δr*poE* and *rpoE* ^+^ strains that were cultivated at various temperatures. The results show the differences relative to the levels observed in the wt strain that was cultured at 22°C. The results are presented as the mean ± S.D. (*n* = 3). *, *P*<0.05, and **, *P*<0.01, based on ANOVA comparisons. (**C**) Western blot assays showing Asp expression in the Δr*poE* and *rpoE* ^+^ strains at different temperatures. RNAP was used as the loading control for the supernatants that were obtained from the same amount of cells.

We then asked which sigma factor governs the temperature-dependent transcription of these virulence factors. RpoE, RpoS, and RpoH have been reported to be involved in thermal responses in vibrios [13,46,47]. Because RpoH is controlled by RpoE activity [15,19], we investigated the roles of RpoE and RpoS in the expression of Asp. The transcription of both sigma factors increased as the temperature of the cultures increases, reaching a maximum expression at 42°C (S2 Fig). Asp promoter (P_*asp*_) activity in a Δ*rpoS* mutant was similar to activity in the wt (Fig 2A). However, in the Δ*rpoE* strain, P_*asp*_ was not upregulated by elevations in temperature (Fig 2A). Similarly, qRT-PCR and Western blot analysis demonstrated that there was a strong reduction in *asp* expression in the Δ*rpoE* mutant, and complementing *rpoE* expression in the Δ*rpoE* strain (*rpoE* ^+^) restored the production of Asp to wild-type levels (Fig 2B and 2C). Taken together, these results demonstrate that RpoE plays a critical role in the temperature-dependent regulation of Asp expression.

### RpoE directs the LuxR- and temperature-dependent expression of virulence factors

**Fig 3:**
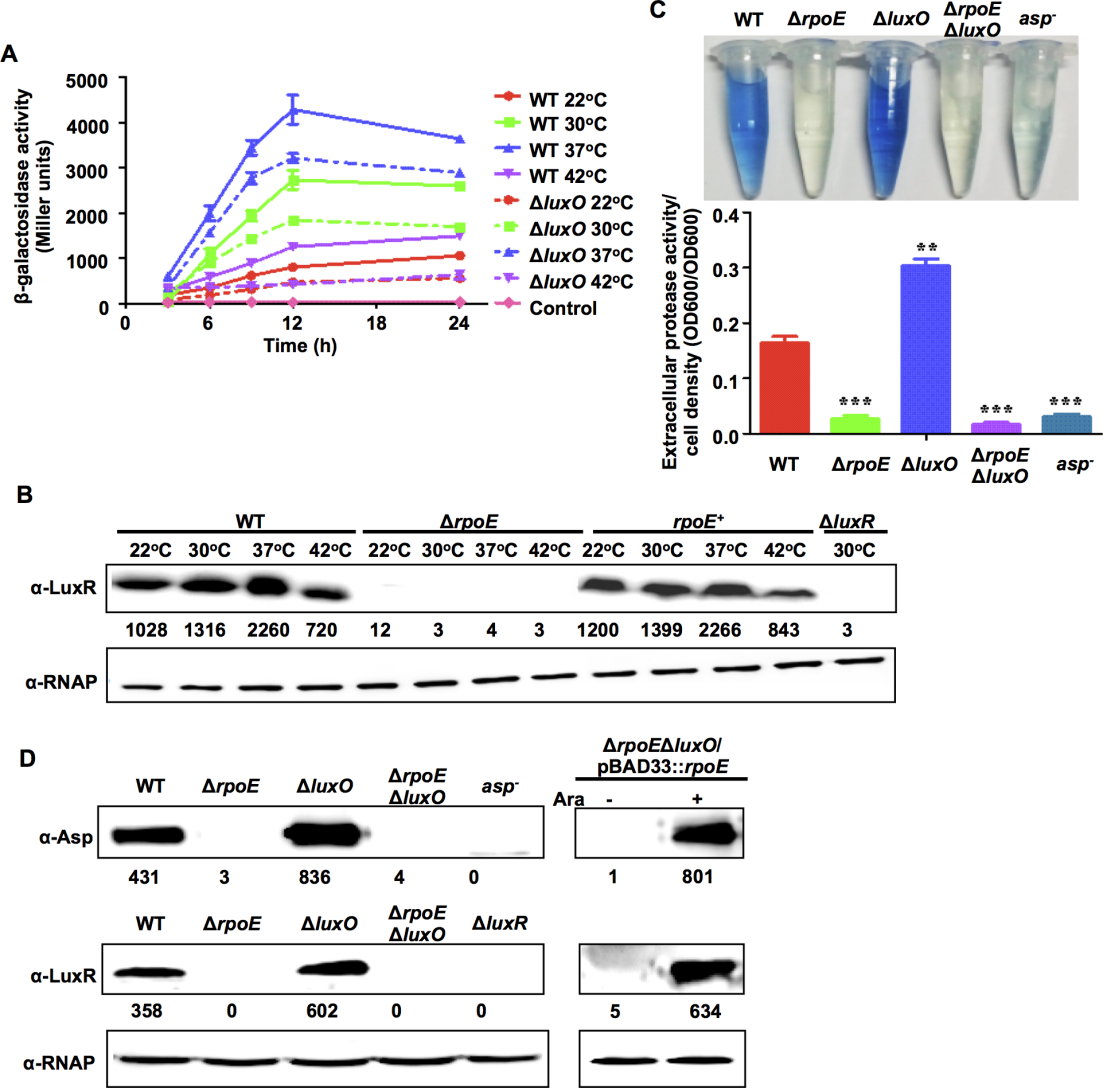
RpoE controls Asp expression via the QS regulator LuxR. (**A**) *luxR* transcriptional assays were performed in wt and Δ*luxO* strains that were grown at different temperatures. The wt and Δ*luxO* strains that carried the P_*luxR*_-*lacZ* reporter plasmid were cultured in LBS medium and assayed for *β*-galactosidase activity. The results are presented as the mean ± S.D. (*n* = 3). (**B**) Western blot assays show LuxR expression in wt, Δ*rpoE* and *rpoE* ^+^ strains grown at different temperatures. Bacteria were cultured in LBS medium for 9 h and then harvested. Proteins from equal cell volumes were resolved using 12% SDS-PAGE. (**C**) Extracellular Asp activity in the *V*. *alginolyticus* strains. The bacteria were centrifuged after they were cultured in LBS medium for 9 h, and the supernatants were harvested to measure protease activity. The results are presented as the mean ± S.D. (*n* = 3). **, *P*<0.01, and ***, *P*<0.001, based on ANOVA comparisons. (**D**) Western blot assays were performed to analyze Asp and LuxR expression in wt and *rpoE-* and *luxO*-related mutants. pBAD33::*rpoE* was introduced into the Δ*rpoE*Δ*luxO* strain to analyze Asp and LuxR expression in the presence (+) and absence (-) of L-arabinose (Ara).

Previous studies have established that LuxR is essential for the expression of Asp and other virulence factors in *V*. *alginolyticus* [36,37]. We found that activity at the LuxR promoter (P_*luxR*_) increased in WT strains as temperature increased from 22°C to 37°C but was lower at 42°C (Fig 3A). LuxR protein levels were also temperature-dependent in the wt cells (Fig 3B). While LuxR expression was completely absent in the Δ*rpoE* mutants (Fig 3B), complementation of Δ*rpoE* with *rpoE in trans* restored the temperature-dependent regulation of LuxR expression (Fig 3B). These results indicate that LuxR expression is controlled by RpoE.

In the QS cascade, LuxO represses the expression of LuxR and Asp. We measured the expression levels of LuxR and Asp in Δ*rpoE*Δ*luxO* double mutants to determine whether the decreased expression of LuxR and Asp in the absence of *rpoE* resulted from an increase in the expression of LuxO. The expression of Asp and LuxR was significantly lower in the Δ*rpoE*Δ*luxO* mutant than in the Δ*luxO* mutant (Fig 3C and 3D). These results contradict the idea that RpoE regulates LuxR expression via LuxO. Furthermore, expressing *rpoE* in the Δ*rpoE*Δ*luxO* and Δ*rpoE* strains restored the expression of both *luxR* and *asp*, demonstrating that *luxR* expression is *rpoE*-dependent and that *rpoE* acts on the LuxR regulatory pathway independent of LuxO and upstream QS cascades (Fig 3B and 3D).

To further explore the dynamics of RpoE-driven *luxR* and *asp* expression, the P_*luxR*_-*lacZ* reporter was introduced into the Δ*luxO* mutant strain to avoid the intervention of QS signaling because it has been established that LuxO acts as a relay in all three of its known signaling cascades [21,24,40]. The results revealed that *luxR* expression remained temperature-dependent and was expressed at lower levels during the early growth phases in Δ*luxO* mutants than in wt specimens (Fig 3A). Low level of P_*luxR*_-*lacZ* expression in the Δ*luxO* mutant could be due to the increase in LuxR production or negative autoregulation as discussed below. Taken together, these results demonstrate that RpoE activates the expression of *luxR* and virulence genes independent of LuxO.

### RpoE binds directly to the *luxR* promoter region to activate its transcription

**Fig 4:**
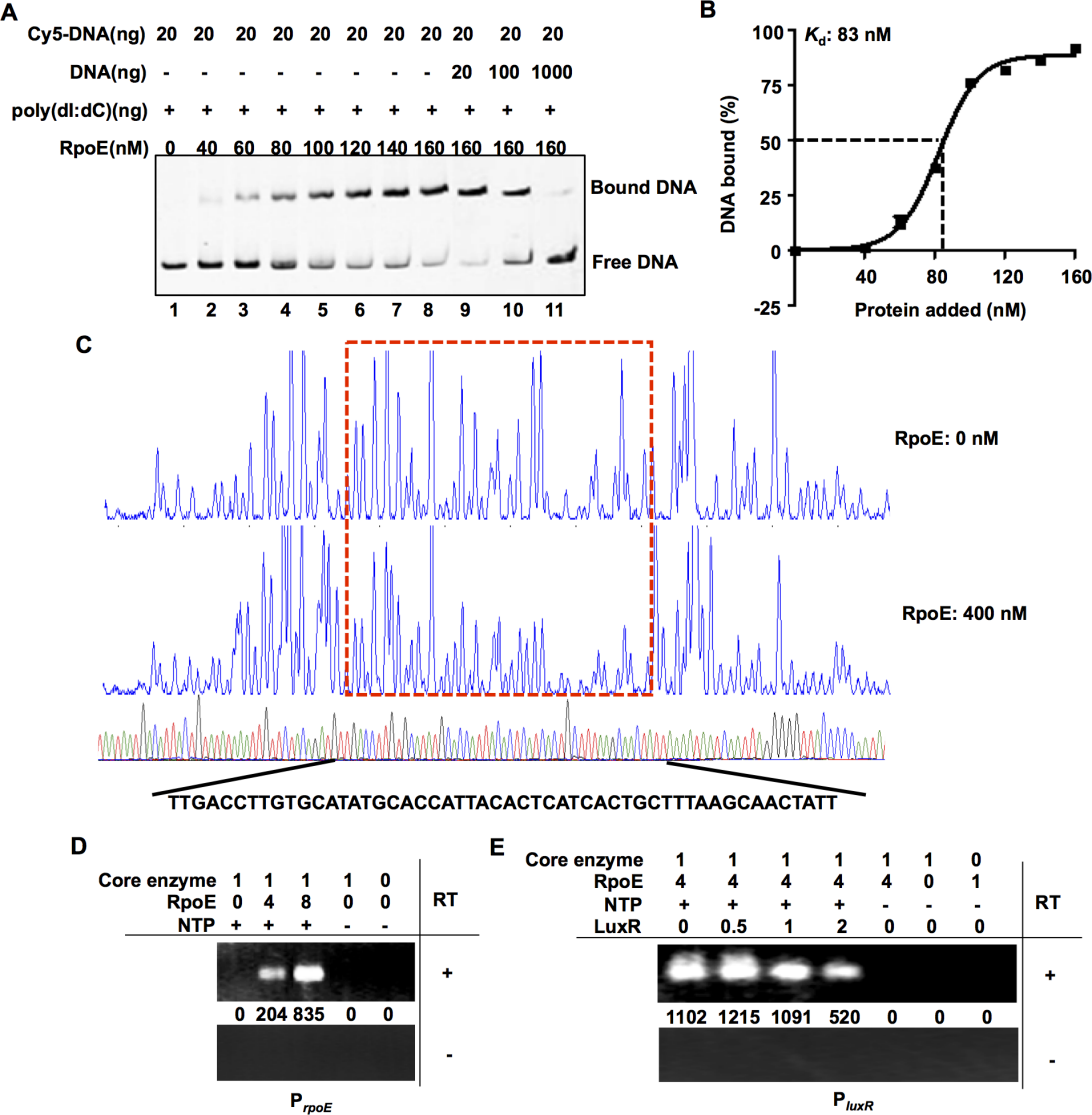
RpoE binds directly to the *luxR* promoter region. (**A**) EMSAs were performed with purified RpoE, and the *luxR* promoter region was analyzed. The amount of RpoE protein (nM) that was used is indicated, and 20 ng of each Cy5-labelled probe was added to the EMSA reactions. The shifts were verified to be specific in experiments in which we added a 5- to 50-fold excess of unlabeled specific DNA and non-specific competitor DNA (poly(dI:dC)). (**B**) Plot showing the affinity of RpoE to the *luxR* promoter. The densitometric intensities of bound DNA fragments were plotted against RpoE concentrations. The arrow indicates the concentration of RpoE that caused half-maximal binding (*K*_d_). (**C**)Footprinting analysis of RpoE binding to a binding site in the *luxR* promoter. Electropherograms of a DNase I digest of the P_*luxR*_ promoter probe (400 ng) after incubation with 0 or 400 nM RpoE. The respective nucleotide sequences that were protected by His-RpoE are indicated below, and the specific -10 and -35 regions are underlined. (**D-E**) *In vitro* transcription was performed using a P_*rpoE*_ template, (**D**) a P_*luxR*_ template (**E**), NTP, and RNAP core enzyme as well as RpoE. Various concentrations of purified LuxR were added into the reaction mixture to determine its effect on *luxR* transcription (**E**). The transcripts were purified, reverse-transcribed (RT, +) and detected using PCR. As a control, the same purified transcripts were also treated using the same process but without reverse transcriptase (RT, -).

Electrophoretic mobility shifts assays (EMSAs) showed that RpoE bound specifically to a DNA probe that included the upstream region of the *luxR* gene (Fig 4A). The *K*_d_ value for RpoE binding to *luxR* was calculated as 83 nM (Fig 4B). The promoters transcribed by RpoE share canonical GGAACTT and TCAAA motifs at locations -35 and -10 relative to the transcription start site [19]. A dye-primer-based DNase I footprint assay was performed on both strands of a DNA fragment that encompassed the entire intergenic region between *luxR* and an upstream gene that encoded a PRTase protein. We compared the electropherograms with and without RpoE, and the results revealed specific RpoE-protected regions that contained the predicted RpoE binding site (TGACCTT for the -35 region and TCATCA for the -10 region) in the *luxR* promoter region (Fig 4C). The absence of a specific binding site abolished the capacity of RpoE to bind to the *luxR* promoter (S3A Fig). To further investigate the *luxR* transcription start site (TSS), we performed a 5’ RACE experiment using RNA that was extracted from wt and Δ*rpoE* strains. The results indicated that there was a specific TSS in the *luxR* promoter region (see below). *In vitro* transcription assays were also performed using the *E*. *coli* RNAP core enzyme and *V*. *alginolyticus* RpoE, *rpoE* or *luxR* genes. The transcripts from various conditions were tested using reverse transcription (RT) PCR with specific primers that targeted the ORF regions. The results indicated that RpoE could functionally and specifically target the *E*. *coli* RNAP core enzyme to transcribe the *rpoE* and *luxR* genes *in vitro* (Fig 4D and 4E). Taken together, these data indicate that RpoE binds to the *luxR* promoter at a distinct site to drive the transcription of *luxR*.

### RpoE binding to the *luxR* promoter is temperature-dependent

**Fig 5:**
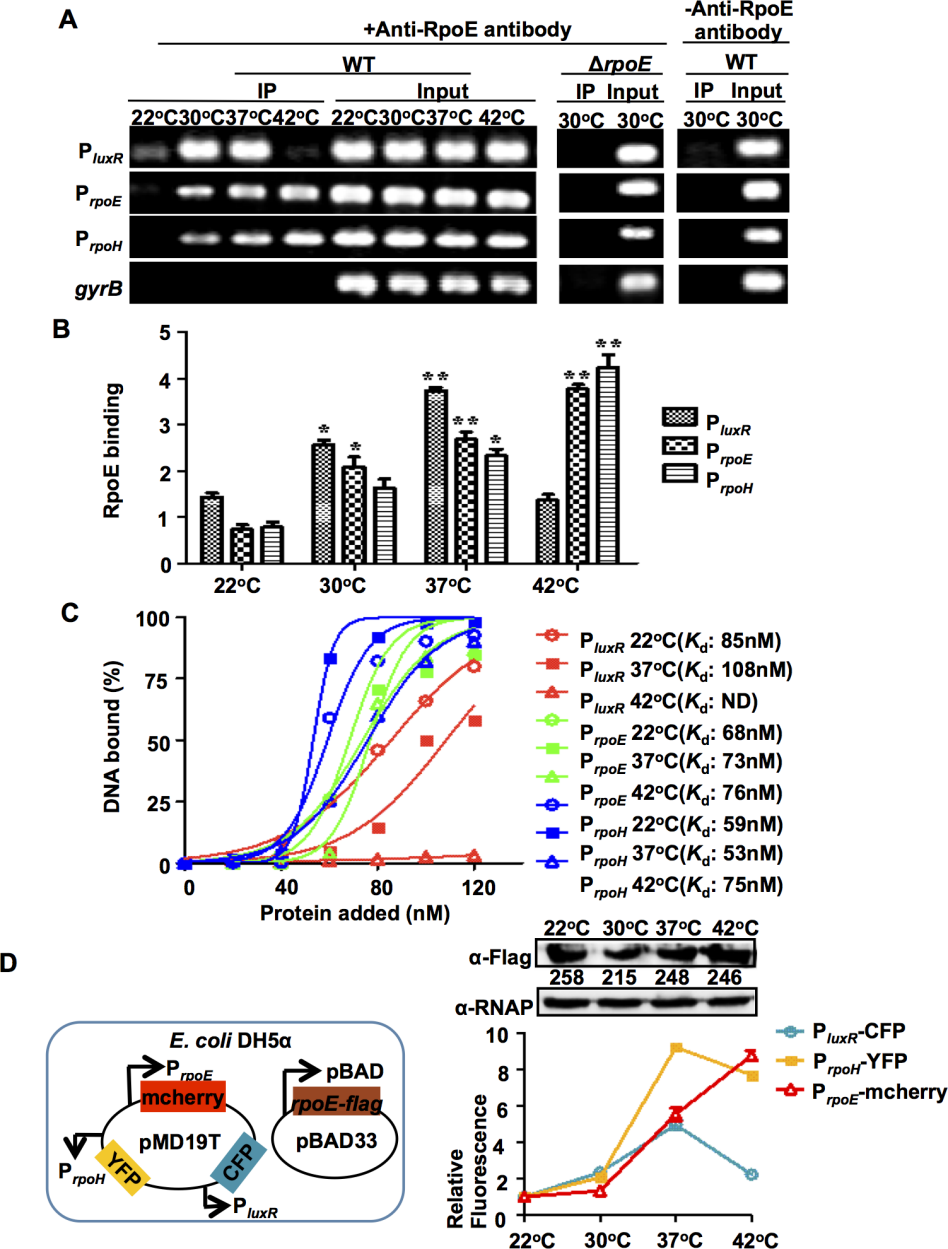
RpoE binds to target DNA in response to temperature changes *in vivo* and *in vitro*. (**A**) ChIP assays were used to analyze RpoE binding to the *luxR*, *rpoE* and *rpoH* promoter *in vivo*. Cells were cultured at different temperatures for 9 h. They were then cross-linked, washed, and sonicated to produce sheared chromosomal DNA was purified from the sheared pellets both before precipitation (input) and after precipitation in the presence (+) and absence (-) of the anti-RpoE antibody (IP). The DNA was then amplified using PCR with the primers P_*luxR*_chip-F/R, P_*rpoE*_-chipF/R, P_*rpoH*_-chipF/R and control-F/R (S2 Table). (**B**) ChIP assays were followed by qPCR to determine the relative enrichment in DNA molecules that were bound to RpoE at different temperatures. The results are shown normalized to the control gene *gyrB*. Results were calculated using the ΔΔ*C*_T_ method. * *P* <0.05, ** *P* <0.01, *t-*test. (**C**) Plot showing the affinity of RpoE binding to the promoters of *luxR*, *rpoE*, and *rpoH* at different temperatures as determined using EMSA (S4 Fig). The intensities of the bound DNA fragments were determined using a densitometer and plotted against the RpoE concentrations. Triplicate assays were performed, and a representative plot is shown. (**D**) The promoter strength of *luxR*, *rpoE*, and *rpoH* in the presence of similar levels of RpoE at different temperatures in *E*. *coli* DH5α cells. The three indicated promoters were fused to different fluorescence reporters and cloned into the same cloning plasmid, pMD19T. The fluorescence reads for each of the promoters after incubation in the presence of arabinose inducing pBAD-driven *rpoE-flag* expression were subtracted from the reads obtained with no arabinose and normalized to both the corresponding reads at 22°C and the densitometry values of RpoE expression. The results are presented as the mean ± S.D. (*n* = 3).

To further investigate the molecular mechanism(s) underlying the RpoE-mediated, temperature-dependent regulation of virulence gene expression, we performed ChIP experiments at different temperatures. Cross-linked DNA obtained from wt and Δ*rpoE* cells was immuno-precipitated using an antibody against RpoE. The input DNA from both the wt and Δ*rpoE* strains were used as controls, and PCR was performed using primers that specifically targeted the region carried an RpoE-binding (RpoEB) site in *luxR*, which generated specific amplification products (Fig 5A). After we reversed the cross-links, the RpoEB site-containing fragment in *luxR* was detected in the immuno-precipitate of wt strains that cultured at various temperatures in the presence of an anti-RpoE antibody. The presence of a PCR product in the immuno-precipitated pellets could be attributed to the specific binding of the RpoE protein to the RpoEB in *luxR* DNA because no PCR product was detected in the IP in the absence of the anti-RpoE antibody (Fig 5A). Consistent with these findings, no *luxR* fragment was detected in the anti-RpoE immuno-precipitate that was obtained from the Δ*rpoE* mutant (Fig 5A). These results verified that the RpoE protein binds directly to the *luxR* regulatory region in *V*. *alginolyticus* both *in vivo* and *in vitro* (Fig 4).

As shown in previous studies [19], RpoE activates the *rpoE* and *rpoH* promoters in response to cytoplasmic thermal stresses. Therefore, we also performed ChIP assays for these two genes using the same IP assay and input samples that we used for *luxR*, and the results confirmed the presence of specific IP products for these two genes at various temperatures (Fig 5A). The enrichment patterns for RpoE binding to the *luxR* promoter region and the *rpoE* and *rpoH* promoters varied at different temperatures (Fig 5A and 5B). At 22°C, there was extremely low or no enrichment for all the three regulatory regions by RpoE. However, when the temperature was increased to 30°C and 37°C, enhanced enrichments were detected in these regions. At 42°C, we detected much lower levels of the P_*luxR*_ region in the precipitated products than at the *rpoE* and *rpoH* promoters. These results suggest that temperature plays a critical role in modulating RpoE expression levels and/or the binding affinity of RpoE to its distinct targets during adaptation to various growth conditions.

We next used EMSA to characterize the *in vitro* binding of RpoE to several of its targets at different temperatures. At 22°C, RpoE bound to all three targets with high affinity (Fig 5C and S4 Fig), suggesting that the lack of or low level of enrichment of the targets *in vivo* (Fig 5A) might have resulted from the low expression of *rpoE* at this temperature (S2 Fig). At 42°C, RpoE exhibited extremely low binding capacity to P_*luxR*_, but its binding affinity for the *rpoH* and *rpoE* promoters remained high (*K*_d_ = 75 nM for P_*rpoH*_, *K*_d_ = 76 nM for P_*rpoE*_) (Fig 5C).

We further characterized the *in vivo* interaction between RpoE and these promoters in *E*. *coli* DH5α cells, which lack QS regulatory circuits (i.e., LuxO and LuxR). We constructed a triple reporter system on a single plasmid that encoded P_*luxR*_ fused to CFP (P_*luxR*_-CFP), P_*rpoH*_-YFP, and P_*rpoE*_-mCherry, and we transformed this triple reporter plasmid along with a second plasmid that over-expressed *rpoE* into the *E*. *coli* cells (Fig 5D). The use of arabinose to induce a high level of plasmid-encoded RpoE minimized the intervention of background low levels of chromosomal RpoE expression. An assay to determine *luxR*, *rpoH*, and *rpoE* promoter activity showed that at 22°C, the activity of these three promoters was relatively low and that it increased by 4- to 9-fold when the temperature was increased to 37°C. However, at 42°C, the activity of the *luxR* promoter was reduced by 2-fold, while the activity of the *rpoH* and *rpoE* promoters remained high, further suggesting that an *in vivo* redistribution or disassociation of RpoE to its different chromosomal binding sites occurs at 42°C that turns off QS regulation, thereby enabling the optimization of cellular responses to thermal stresses in *V*. *alginolyticus*.

### Interplay between RpoE, LuxR and AphA modulates *luxR* expression

Because LuxR and AphA are known to be *luxR* regulators in other vibrios, and because AphA is an established repressor of *luxR* expression [24,27], we next investigated whether the RpoE-mediated control of *luxR* transcription was modified by these transcription factors. Inactivating *luxR* and *aphA* resulted in ~1.5-fold increase in P_*luxR*_ expression in the stationary phase. However, in the Δ*rpoE*Δ*luxR* and Δ*rpoE*Δ*aphA* double mutants, P_*luxR*_ activity was completely abolished (S5A Fig). *In vitro* transcription assays using the *E*. *coli* RNAP core enzyme showed that LuxR repressed the production of *luxR* transcripts (Fig 4E). Collectively, these data suggest that LuxR and AphA function as repressors of *luxR*, whereas RpoE is essential for its expression.

**Fig 6:**
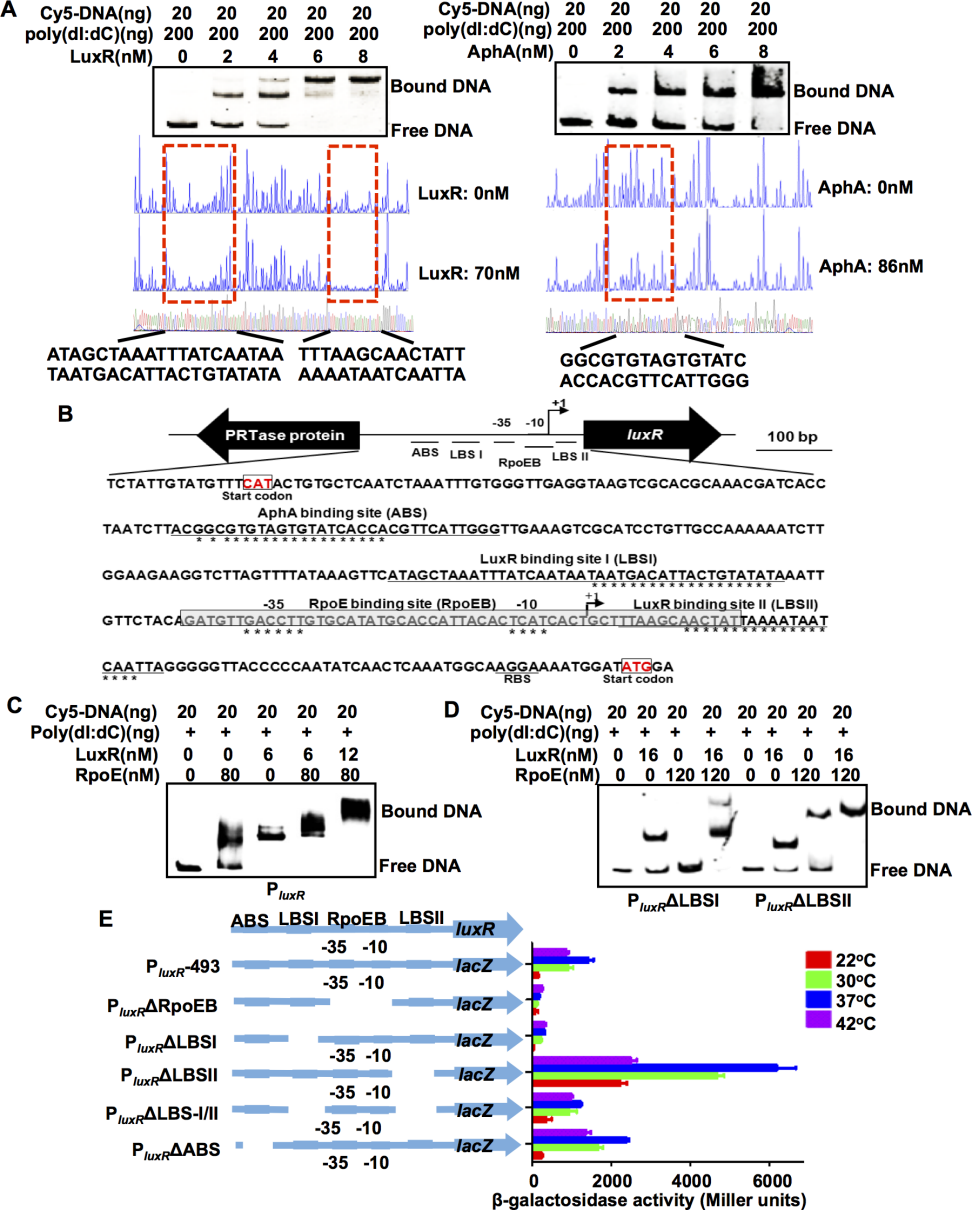
RpoE, AphA, and LuxR bind to the *luxR* promoter. (**A**) EMSA and DNase I footprinting analysis were used to analyze whether LuxR and AphA specifically bind to the *luxR* promoter. In EMSA assays, various concentrations of LuxR and AphA proteins were added to mixtures of poly(dI:dC) and Cy5-labeled *luxR* promoter DNA. In the DNase I footprinting assays, electropherograms of a DNase I digest of the P*luxR* promoter probe after it was incubated with or without the LuxR and AphA proteins. The level at which the respective nucleotide sequences were protected by the proteins is indicated below. (**B**) Diagram the showing promoter region of the *luxR* gene. The LuxR and AphA binding sites and the ribosome binding site (RBS) are underlined. The region protected by RpoE (RpoEB) is shadow-boxed. The well-conserved nucleotides that were bound to specific proteins are indicated with asterisks. The transcription start site (TSS) is labeled as +1. (**C-D**) EMSA assays of LuxR and RpoE mixtures binding to *luxR* promoter or mutant *luxR* promoter regions in the absence of the LBSI (P_*luxR*_ΔLBSI) or LBSII binding site (P_*luxR*_ΔLBSII). The amounts of LuxR or RpoE protein (nM) that were used are as indicated, and 20 ng of each Cy5-labelled probe was added to the EMSA reactions. (**E**) The promoter activity is shown for P_*luxR*_ and its variants that were fused to *lacZ*. The strains were grown at 22°C, 30°C, 37°C and 42°C for 9 h, and *β*-galactosidase activity was then assayed. The results are shown as the mean ± S.D. (*n* = 3).

The binding of LuxR and AphA to P_*luxR*_ was then tested so that we could begin to decipher the mechanisms by which binding by these negative regulators of *luxR* expression interacts with RpoE binding. A 298-bp DNA fragment encompassing the *luxR* promoter region was incubated with increasing amounts of LuxR and/or AphA and then subjected to EMSA. We observed that the P_*luxR*_ fragment specifically bound to LuxR and AphA. The addition of LuxR resulted in a concentration-dependent ladder of two slowed bands, suggesting that there are at least two binding sites with different affinities for LuxR within the P_*luxR*_ region (Fig 6A). In similar DNA binding assays, AphA also displayed specific binding to the P_*luxR*_ region (Fig 6A). When both proteins were included in the binding reaction, they bound simultaneously to their operators, which resulted in a more slowly migrating band (S5B Fig).

We preformed DNase I protection assays to map the binding sites for LuxR and AphA in the P_*luxR*_ region. The results revealed two distinct LuxR binding sites (LBSI at -94 to-54 and LBSII at +30 to +3 relative to the TSS) and one AphA binding site (ABS at -256 to -227 relative to the TSS) in this region (Fig 6A and 6B). Because the sequence of the LuxR binding site II (LBSII) extended into the RpoE binding region within P_*luxR*_, it is expected to prevent RNAP binding to and the transcriptional initiation of *luxR*. These results are therefore consistent with our finding that LuxR acts as a repressor of *luxR* expression (S5A Fig).

Additional gel shift assays were performed to further characterize the interplay between LuxR or AphA with RpoE at P_*luxR*_. EMSAs conducted using the LuxR protein and P_*luxR*_ΔLBSI/II, P_*luxR*_ΔLBSI, or P_*luxR*_ΔLBSII revealed that both the LBSI and the LBSII binding site contributed to LuxR binding to P_*luxR*_ (S3B Fig and Fig 6D). When both RpoE and LuxR were present in the P_*luxR*_ binding reaction, most of the bands representing DNA that was bound to either RpoE or LuxR disappeared, and a new and more slowly moving band was observed that represented DNA that was bound to both of the proteins (Fig 6C). These data suggested that RpoE and LuxR can simultaneously bind to the *luxR* promoter region and that there are some cooperative interactions in their binding to the P_*luxR*_ DNA. Binding reactions between LuxR/RpoE and P_*luxR*_ΔLBSI or P_*luxR*_ΔLBSII probes further revealed that these two proteins cooperatively interact when they bind to their corresponding LBSI and RpoEB sites, respectively (Fig 6D). The results also unexpectedly indicated that the LBSI site in P_*luxR*_ is essential for RpoE binding to P_*luxR*_ and that LuxR binding to LBSI can facilitate RpoE-P_*luxR*_ binding (Fig 6D). However, the binding patterns for AphA/RpoE were similar to the profile of either RpoE or AphA alone (S5C Fig), suggesting that there are no cooperative interactions between AphA and RpoE.

We further characterized the roles of the binding sites in the *luxR* promoter region in *luxR* expression at various temperatures. As expected, promoter activity was abolished in the constructs with deletions in the RpoE binding box when strains were incubated at various temperatures (Fig 6E). Similar results were observed in strains with deletions in LBSI, indicating that these two *cis*-acting elements are essential to the activation of *luxR* promoter (Fig 6E). Eliminating the other LuxR binding box (LBSII) resulted in the strong derepression of *luxR* expression (Fig 6E). Eliminating both LBSI and LBSII also resulted in the derepression of *luxR* expression, but to a lesser extent than the mutation of LBSII alone (Fig 6E). These observations reveal that LuxR binding to its promoter has both activating and repressive effects: LuxR binding to LBSII represses transcripts, but binding to LBSI is essential for *luxR* transcription. There was also a marked increase in P_*luxR*_ activity in strains in which the AphA binding site was deleted (P_*luxR*_ΔABS). In terms of temperature regulation, in nearly all cases, 37°C, among all of the temperatures that were tested, was the optimal temperature for boosting the expression of various P_*luxR*_ constructs. The exception was strains lacking the RpoE binding box (P_luxR_ ΔRpoEB) and LBSI (P_*luxR*_ ΔLBSI), which suggests that the elements discussed above might not be involved in sensing or responding to temperature variations. A *luxR* promoter construct lacking both binding sites of LuxR (P_*luxR*_ΔLBS-I/II) was expressed similarly at 30°C, 37°C and 42°C (Fig 6E). Removing the binding of LuxR to *luxR* promoter region might result in the changes in the RpoE occupancy in *luxR* promoter, and thus alter the temperature-dependent affinity and binding dynamics of RpoE-P_*luxR*_ DNA interaction. Collectively, these data support the idea that *luxR* expression is controlled by a complex interplay between RpoE, LuxR and AphA and the notion that LuxR and RpoE are required for *luxR* expression, while the former can, like AphA, also act as a repressor.

### Anti- σ^E^ signaling is required for the temperature induced QS cascade

**Fig 7:**
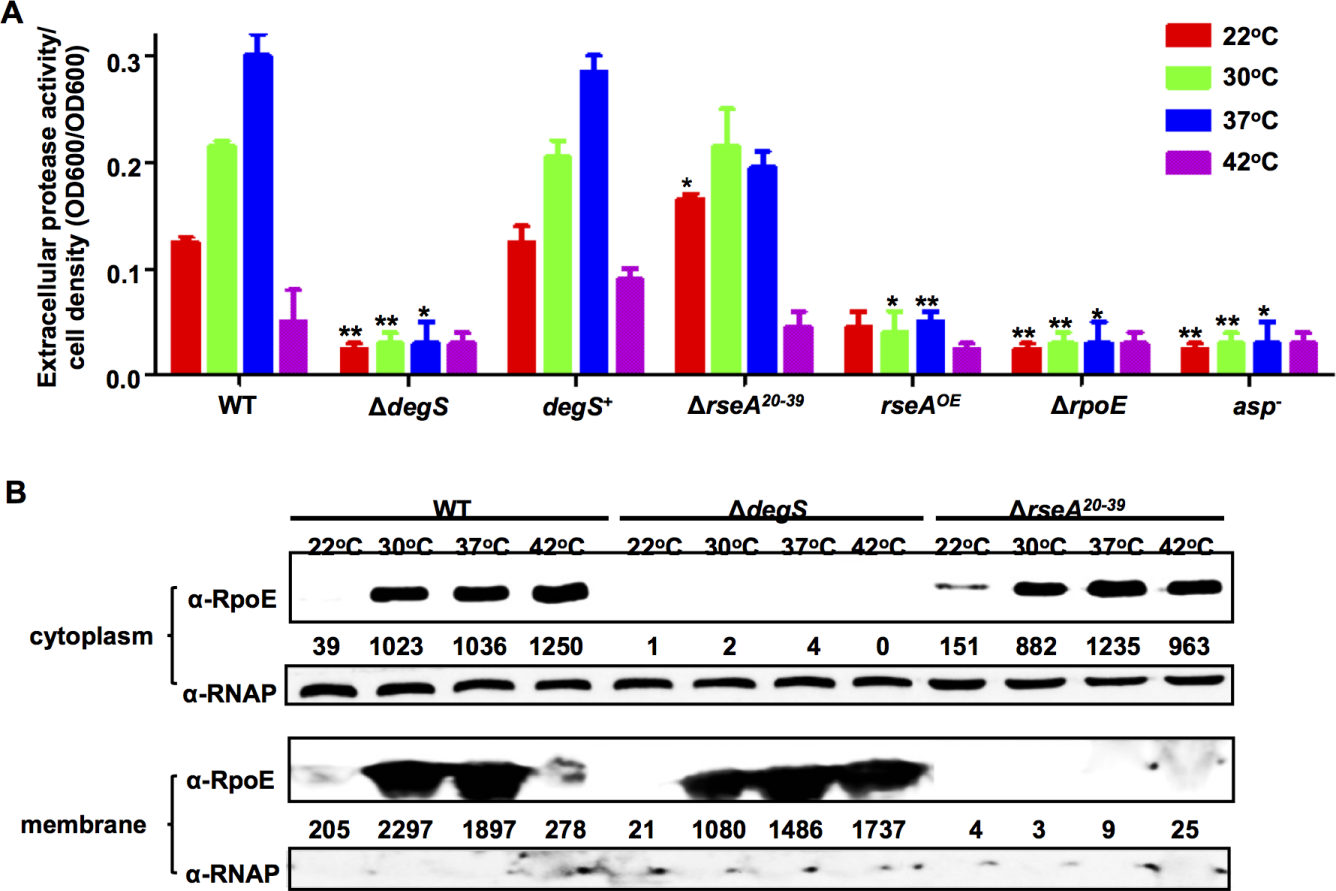
RIP and anti-sigma E signaling pathways are involved in RpoE-regulated QS. (**A**) HPA digestion assays were performed to analyze extracellular Asp activity in wt and mutant strains. Bacteria were centrifuged after they were cultured in LBS medium for 9 h at various temperatures. The supernatants were harvested, and protease activity was measured. ECP activities were normalized by dividing the total level of activity by the cell density for each strain. The results are shown as the mean ± S.D. (*n* = 3). * *P* <0.05, ** *P* <0.01, *t-*test compared to the corresponding results in the wt strain at each temperature. (**B**) Western blot analysis was performed to determine the distribution of RpoE in membrane and cytoplasmic pellets in wt, Δ*degS* and Δ*rseA* ^20-39^ strains at different temperatures. Bacterial cells were centrifuged after they were cultured in LBS medium for 9 h, and the cytoplasmic and membrane protein pellets were then extracted. RNAP was used as a loading control.

Under normal conditions, RseA binds σ^E^ to the inner membrane. During envelope stress conditions, RseA is cleaved by DegS and RseP, which leads to the release of σ^E^ [48] and is an essential step to control the activity of RpoE [48]. The operon *rpoE*-*rseABC* is highly conserved among *V*. *alginolyticus*, other vibrios and *E*. *coli* (S6A Fig). In bacterial two-hybrid assays, *V*. *alginolyticus* RseA interacts with RpoE (S6B Fig–S6D Fig). We investigated whether RseA and the related components of the signaling cascade that leads to the activation of RpoE were required for the RpoE- and temperature-dependent control of the *V*. *alginolyticus* QS. As shown in Fig 7A, in a Δ*degS* mutant, the expression of Asp was reduced to the same levels that were observed in the *rpoE* and *asp* mutants, and complementation with *degS* restored expression to wt levels. These results are consistent with the observation that the release of RpoE from the membrane was blocked in the Δ*degS* mutant (Fig 7B), which resulted in decreased Asp expression.

The Δ*rseA* ^*20-39*^ strain, in which a region of the N-terminal domain (aa 20–39) that is required for RseA to tether RpoE to the membrane [18] (Fig 7B) has been deleted, displayed dramatically higher levels of Asp production than the RseA overexpressing strain *rseA* ^*OE*^ at temperatures of 22°C to 37°C (Fig 7A), indicating that RseA is involved in RpoE-mediated QS regulation under temperatures ranging from 22°C to 37°C allowing high levels of Asp production (Fig 7A), However, when the temperature was increased to induce thermal stress conditions (42°C), the Δ*rseA* ^*20-39*^ strain produced high levels of cytoplasmic RpoE (Fig 7B) and marginal levels of Asp (Fig 7A).Taken together, these data indicate that anti- σ^E^ signaling is essential for the switch from a QS cascade to thermal stress adaptation in *Vibrio alginolyticus*.

### RpoE plays essential roles in *V*. *alginolyticus* responses to various stresses and in its temperature-dependent virulence in fish

RpoE plays an important role in stress tolerance in many pathogens. The survival rates in wt, Δ*rpoE*, and *rpoE* ^+^ specimens were determined using CFU counts after the cells were exposed to diverse stress conditions, including sucrose (S7A Fig), ethanol (S7B Fig), heat (S7C Fig), H_2_O_2_ (S7D Fig), and NaCl (S7E Fig and S7F Fig). After exposure to all of these stresses, the Δ*rpoE* mutant exhibited significantly lower survival rates than the wt and *rpoE* ^+^ complemented strain. These results indicate that RpoE contributes to the ability of *V*. *alginolyticus* to adapt to various stresses.

**Fig 8:**
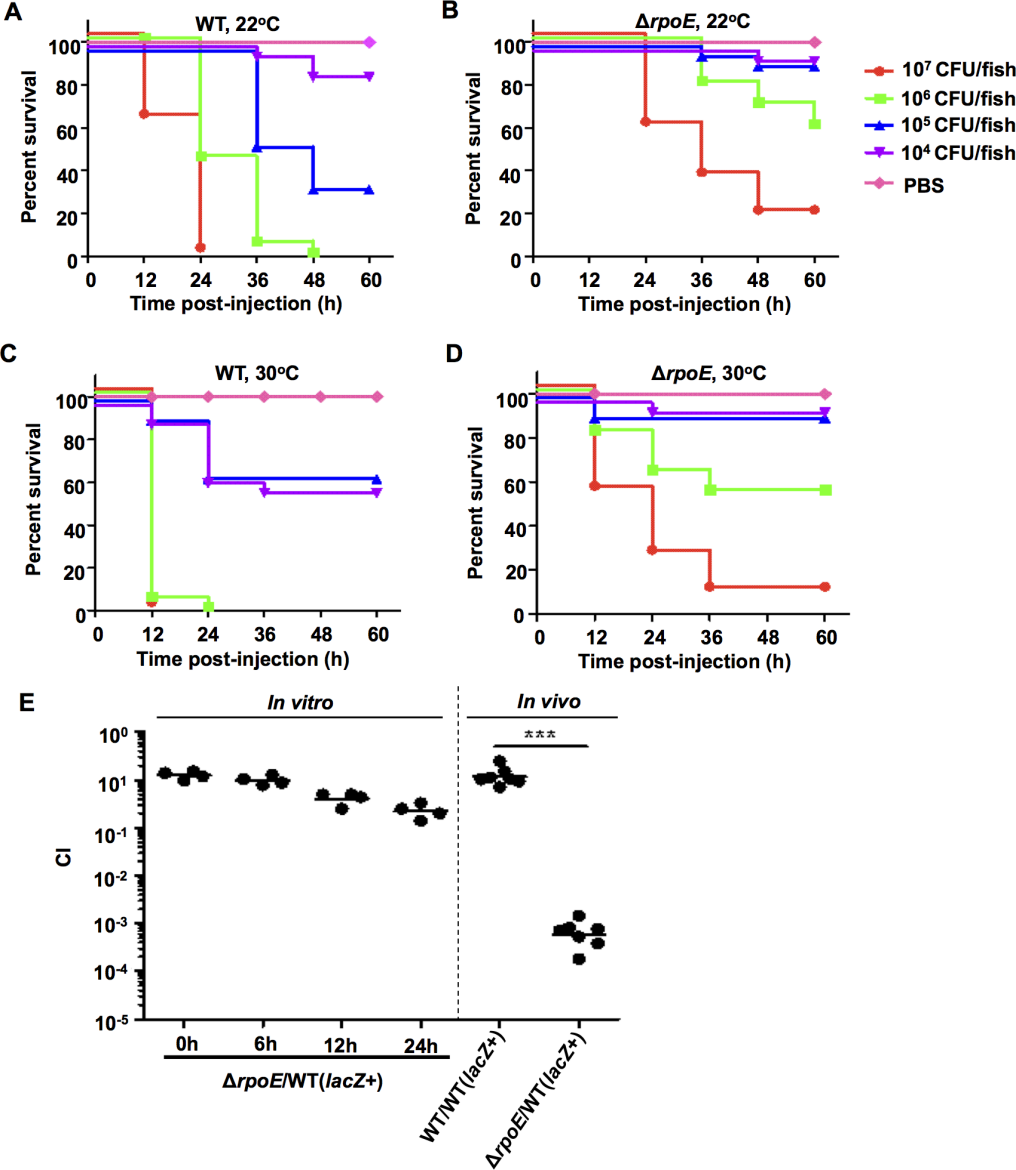
RpoE is involved in the temperature-dependent *in vivo* virulence of *V*. *alginolyticus*. (**A-D**) Virulence was analyzed in the *rpoE* mutant in zebrafish. A series of dilutions of the wt and *rpoE* mutant strains were intramuscularly (i.m.) inoculated into fish that were acclimated at 22°C or 30°C for 4 weeks. A total of 30 fish were used for each of the dilutions. The infected fish were cultivated at 22°C or 30°C and monitored for 7 days. *P* values were calculated using Kaplan-Meier survival analysis with a log rank test. (**E**) CI assays for the Δ*rpoE* strain against the wt (*lacZ* ^+^), which was a wt strain carrying *lacZ* following the *glmS* locus. The CI values of the 1:1 Δ*rpoE* vs. wt (*lacZ* ^+^) inoculums were first tested in LBS medium at 30°C for 24 h. Then, 1:1 [the indicated strains vs. wt (*lacZ* ^+^)] inoculums were i.m. administered into zebrafish and cultivated at 30°C for 24 h before the fish were anesthetized and their cells were numerated on IPTG plates. ***, *P*<0.001, based on ANOVA followed by Bonferroni’s multiple-comparison post-test to compare the data to the values for the corresponding wt/wt (*lacZ* ^+^) samples.

Zebrafish was used as a model system to test the impact of RpoE and temperature on the virulence of *V*. *alginolyticus*. In all of these experiments, groups of zebrafish (*n* = 30) were acclimated to distinct temperatures (22°C and 30°C) for at least 4 weeks before the infection experiments were performed. No apparent stress or disease symptoms were observed when the fish were cultivated at these two temperatures than when they were cultivated at a normal breeding temperature of 28°C [49]. At 22°C, the group of fish inoculated with a high dose (10^7^ CFU/fish) of wt *V*. *alginolyticus* died rapidly within 24 h, while the fish treated with the same dose of the Δ*rpoE* mutant displayed a 26.7% survival rate after 60 h of observation (Fig 8A and 8B). All of the dead fish exhibited typical hemorrhagic septicemia. A marked difference (*P*<0.0001) in the survival rates was observed between the groups of fish that were treated with wt (16.7%) and Δ*rpoE* strain (86.7%) at a dose of 1×10^6^ CFU/fish, as well with 10^5^ and 10^4^ CFU/fish (Fig 8A and 8B). When the animals were raised at 30°C, a 100% rate of fish death was observed following infection with the high dose (1×10^7^ and 1×10^6^ CFU/fish) of wt *V*. *alginolyticus*, while much higher survival rates (16–59%) were observed in the fish that were infected with the Δ*rpoE* mutant (Fig 8C and 8D). Significant differences (*P*<0.001) were also observed in survival rates between the groups of fish that were inoculated with wt *V*. *alginolyticus* and Δ*rpoE* at doses ranging from 10^5^−10^4^ CFU/fish (Fig 8C and 8D). The LD_50_ values of the Δ*rpoE* and the wt were 4.2×10^6^ and 2.7×10^5^ CFU/fish at 22°C, and 2.6×10^6^ and 1.6×10^4^ CFU/fish at 30°C, respectively, showing that temperature modulates the severity of virulence in the *rpoE* deletion mutant in *V*. *alginolyticus*. We also observed a profound competitive defect in the *in vivo* growth (>1000-fold) of the *rpoE* mutant in comparison to the wt strain when the two strains were equally co-inoculated into zebrafish, although there was a slight competitive defect (~10-fold) in *rpoE* mutants grown in LBS after 24 h of co-culture with the wt strain (Fig 8E). Collectively, these data reveal that RpoE exerts potent and temperature-dependent control over *V*. *alginolyticus* pathogenicity in zebrafish.

## Discussion

### Common and distinct strategies are employed to regulate virulence in response to temperature increases in *Vibrio* spp.

Temperature is a critical and ubiquitous environmental signal that governs the development and virulence of diverse microbial species. The science of systematically unraveling the mechanisms underlying the temperature-mediated regulation of virulence is still in its infancy. As the most abundant flora in marine environments [3,8], *Vibrio* spp. must have evolved a wide variety of molecular mechanisms through which cells sense and respond to alterations and fluctuations in the temperature of the ocean [50,51]. Vibrios, including *V*. *cholerae*, *V*. *parahaemolyticus*, *V*. *alginolyticus*, and *V*. *vulnificus*, are also prevalent and notorious zoonotic pathogens in sea animals and humans [31]. It is therefore not uncommon for these species to possess a repertoire of strategies to orchestrate cellular signaling, developmental programs, and pathogenesis so that they can respond to or even leverage signal shifting in response to higher temperatures (e.g., 30°C -37°C), for the population to thrive in hostile environmental conditions and host milieus, enabling them to cause diseases in various animal hosts, including coral, fish, shellfishes and humans. In this study, we present a novel temperature-responsive paradigm that includes RIP and σ^E^-anti σ^E^ signaling and their integration into QS circuits that optimizes virulence production and stress adaptation in the zoonotic pathogen *V*. *alginolyticus*.

In the present study, we found that upshifting temperatures increased the expression and production of exotoxins while the expression of these virulence factors was dramatically decreased under thermal stressed temperatures (e.g., 42°C) in the zoonotic pathogen *V*. *alginolyticu*s (Fig 1). In other independent reports, *V*. *alginolyticus* has also been observed to regulate the production of ECPs in a temperature-dependent manner [52,53], although a variety of ECPs have been observed in different strains, and ECP production dynamics are different in different strains [34,52,53]. A previous investigation also indicated that in this bacterium, swimming and swarming motility were modulated at different temperatures as observed in our strain (S7G Fig and S7H Fig) [43]. These results collectively suggest that the mechanisms by which *V*. *alginolyticus* senses temperature shifts and regulates the production of virulence-associated factors might be conserved or different among various strains. In a previous study by Kimes et al., increasing the temperature from 24°C to 27°C upregulates a large portion (~150) of virulence factors known to be involved in motility, hemolysis, and cytotoxicity in *V*. *coralliilyticus* independent of cell abundance. This organism has been associated with global coral diseases [8]. In particular, the ECPs that were upregulated in this bacterium during the transition from 24°C to 29°C have been shown to be essential for the bleaching and lysis of the coral *Pocillopora damicornis* [54]. In *V*. *vulnificus*, a heme receptor protein, HupA, was found to be specifically induced at temperatures higher than 40°C, demonstrating that in this bacterium, temperature controls the expression of virulence-related factors, specifically the iron uptake system, via an unknown mechanism [9]. In *V*. *cholerae*, it is reported that an RNA thermometer was involved in controlling *toxT* expression, providing evidence that temperature-mediated mechanisms control virulence gene expression in this bacterium [10]. Taken together, vibrios appear to have evolved distinct, but largely undefined, temperature response mechanisms to regulate virulence gene expression.

Proteinaceous regulators, including repressors, anti-repressors, protein chaperons, two-component systems (TCSs), the DNA topology modulator HNS, and RNA thermometers, have been shown to regulate responses to temperature changes in bacteria [51]. Here, our data indicate that RpoE signaling, including the anti-sigma factors RseA and the RIP pathway, are involved in the regulation of QS-controlled virulence gene expression in *V*. *alginolyticus* (Figs 3 and 7). RpoE specifically binds to the promoter region of the QS regulator LuxR to activate the expression of related virulence genes (Fig 4). Because RpoE and *luxR* promoter region are highly conserved in vibrios, and typical P_*luxR*_ from *Harveyi* clade, e.g. *V*. *coralliilyticus*, appears to bind directly to RpoE (S8 Fig), we hypothesize that other related *Vibrio* taxa might also share the same temperature-dependent QS activation mechanisms. These findings suggested that vibrios can manipulate their responses to temperature stimuli and their membrane integrity surveillance machinery by modulating RpoE signaling, thereby enabling cells to rewire gene expression at the population level. As for the effect of temperature on QS regulation, the general idea is that temperature can affect AI production in terms of biosynthesis rates and AI species [11,55–57]. Kimes *et al*. proposed a direct effect of temperature on QS in *V*. *coralliilyticus* that upregulated the expression of virulence factors via an unknown mechanism [8]. Our data (S8 Fig) suggesting that this bacterium, a close relative of *V*. *alginolyticus* in the *Harveyi* clade, can also use a similar RpoE-dependent temperature gauge to regulate QS and cause infections in corals in response to increasing SST.

### Activation of σ^E^ signaling pathway by increasing temperatures potentiates virulence gene expression and switching to thermal stress adaptation

Our data indicate that RpoE is required for optimal growth and adaptation to various stresses, as well as for virulence in *V*. *alginolyticus* (S1 Fig, S7 Fig and Fig 8). In *E*. *coli*, *V*. *cholerae* and other bacteria [16,19,58,59], RpoE controls the expression of a large, conserved set of genes that are functionally related to membrane and LPS integrity (e.g., *ompA*, *ompU*, *ompW*, etc.), thus is essential in these bacteria. In *V*. *cholerae*, *ompU* could be the sole suppressor mutation required for the isolation of a viable *rpoE* mutant [19]. In the present study, we sequenced *ompU* gene (including its promoter and entire ORF) from several (n = 13) colonies of Δ*rpoE* mutant and found that there was no *ompU* mutation present in these Δ*rpoE* mutant colonies, suggesting that *ompU* is not be the suppressor of *rpoE*, as described in the closest relatives *V*. *parahaemlyticus* [14], or is not be responsible for the σ^E^-activation in this bacterium. It is worth noting that although *rpoE* is essential for thermal stress response in *V*. *alginolyticus* (S1B Fig and S7C Fig), high number (~1%) of cells remains viable as quantified with plate counting at 42°C (S1A Fig). Such high number cells allowed us to carry out quantitative assays of *asp* and *luxR* expression after normalization (Figs 2 and 3).

In *E*. *coli*, σ^E^ activity is governed by the rate of degradation of its anti-σ factor, RseA. The DegS protease can initiate RseA cleavage only when DegS is activated by binding to unfolded or misfolded outer-membrane porins (OMPs) [17,48]. OMPs activate the protease DegS, and they also generate a signal to antagonize the inhibition of RseB [60]. Peptides ending with OMP-like C-terminal sequences (YYF-COOH, YQF-COOH or YQM-COOH) can bind the DegS PDZ domain to activate the cleavage of RseA. In *E*. *coli*, both OmpC and OmpF have C-terminal YQF sequences that can potentially bind DegS to activate the degradation of RseA [48]. In *V*. *cholera*, the conformation of OmpU is indispensable to triggering a DegS-dependent σ^E^-activating cascade [14]. Our results showed that the RpoE regulation of LuxR and Asp also depends on the cleavage of RseA by DegS (Fig 7). The question remains as to whether OmpU or other porins/molecules are serving as specific thermal sensors in the RIP-induced activation of RpoE in *V*. *alginolyticus* when temperatures increase.

Transcriptomic analysis has indicated that broad-range of organism-specific functions, including optimal host interactions and pathogenesis are controlled by *rpoE* in various bacteria. We were most interested in *luxR*, which has been shown to be involved in the QS regulation and virulence [36,37]. Indeed, LuxR or QS has been proved to control expression of large set (~600) of genes, including the genes encoding toxins (Asp, Pep, MviN as demonstrated in *V*. *alginolyticus*), motilities, extracellular polysaccharides (EPS), biofilm formation, type III and VI secretion systems (T3SS and T6SS) and other functions [25,36–39,42]. Regulation of LuxR by RpoE seems to further expand the modulon at favorable temperatures in the bacterial population level.

It is intriguing to explore the mechanisms driving the switch from virulence gene expression to thermal stress adaptation at unpermitted high temperatures in *V*. *alginolyticus*. With various *in vitro* and *in vivo* assays (e.g. ChIP-qPCR, EMSA and fluorescence reporters) (Fig 5), we propose that redistribution of RpoE to its different chromosomal binding sites occurring at 42°C turns off QS regulation, thereby enabling the optimization of cellular responses to thermal stresses in *V*. *alginolyticus*. This higher temperature redistribution of RpoE to different binding motifs could attribute to i) the changes of binding specificity of the RpoE-binding motif pairs, and/or ii) increased expression of RpoE dependent activators/modulators for various promoters at thermal-stressed temperatures. The disassociation of RpoE from *luxR* promoter region at 42°C and thus shutting-down virulence gene expression might be resulted from both of the above-mentioned mechanisms. We have demonstrated a lower affinity of RpoE-P_*luxR*_, as compare to RpoE-P_*rpoH*_ and RpoE-P_*rpoE*_ pairs (Fig 5A and 5C), while the possibility that increased expression of RpoE-dependent modulators that may stabilize the specific binding of RpoE to *rpoH* or *rpoE* remains to be further determined. As RpoE binding to *luxR* promoter region seems to be dependent on the LuxR binding to LuxR binding site I (LBSI) (Fig 6E), decreased stability of LuxR binding to LBSI at higher temperatures (e.g. 42°C) might also contribute to the redistribution of RpoE binding to *luxR*. RpoE binding to *rpoH*, *rpoE* or other specific genes involved in canonical thermal stress response pathway might have been evolutionally optimized in terms of binding specificity at higher temperatures [19–20]. While in *V*. *alginolyticus* and other related vibrios, it seems that “recognition” of *luxR* promoter by RpoE is a recently acquired capability to integrate into the virulence regulation pathway, representing as an extended regulatory function of RpoE. It is evolutionally and biologically reasonable that the binding specificity and related “binding modulator” has been fine-tuned for thermal stress cascades and fitness while not for QS regulation at thermal stressed temperatures.

### A sophisticated mechanism for the regulation of *luxR* expression by RpoE, AphA and LuxR

*V*. *alginolyticus* LuxR is a founding member of a TetR family of homologous proteins (e.g., OpaR, SmcR, VanT, HapR, LuxR, and LitR) that control QS known as MQSR in different *Vibrio* species [25]. As cell population densities increase, the AIs accumulate to a level that can be detected, and this ultimately leads to the production of MQSR, which globally controls the expression of hundreds of genes in vibrios. The timing of MQSR expression is therefore the key to obtain protein concentration gradients that function in the temporal regulation of gene expression at different cell densities. To avoid MQSR expression runaway and protein bursts under unsuitable conditions (e.g., low cell densities, unfavorable nutrient status, low temperatures or unpermitted high thermal stress conditions), which would confer a potentially large fitness cost, MQSR production is likely to be strictly controlled at multiple levels so that QS regulon gene expression profiles can be more precisely controlled according to environmental conditions [25]. Increasing number of studies in *V*. *cholerae* has shown that various *hapR* regulators function at the transcriptional level (e.g., Fis, VqmA, HapR, AphA, VarS/VarA, and FliA/FlgM) [27,28,55,61–64], the post-transcriptional level (e.g., *qrrs* and *csrA*-associated sRNAs) [65,66], and the post-translational level (e.g., VarS/VarA) [63], and that these factors are deployed to regulate the production or functions of HapR. These results demonstrate the presence of a multiple-level regulatory network that controls the spatio-temporal production and activity level of MQSR. Interestingly, most of these regulators are repressors of HapR, indicating that a sophisticated network is required for optimal HapR regulation in *V*. *cholerae*.

In the current study, we have provided evidence showing that RpoE and related RIP signaling are required to activate *luxR* transcription in *V*. *alginolyticus* (Figs 3 and 7). Further analyses indicated that RpoE binds to the consensus box in the *luxR* promoter both *in vitro* and *in vivo* and that it targets the RNAP core enzyme to transcribe *luxR* (Figs 4 and 5). These data demonstrate that RpoE is a sigma factor that is responsible for the initiation of *luxR* transcription. Because there is evidence that the binding box and RpoE are conserved in vibrios, especially within the *Harveyi* clade, and that RpoE from *V*. *alginolyticus* can bind to promoter regions in other *Vibrio* promoters (S8 Fig), we anticipated that the RpoE transcriptional dependency of *luxR* expression would be conserved in other vibrios. In a previous study, Chatterjee et al. reported that *E*. *coli* σ^70^-based RNAP binds to the *V*. *harveyi luxR* promoter region [67]. Our study did not exclude the possibility that *luxR* can also be transcribed by σ^70^-based RNAP in *V*. *alginolyticus*. In the *luxR* promoter region, σ^70^-dependent -10 and -35 regions were predicted by an *in silico* analysis. These predicted locations overlapped with the RpoE specific -10 and -35 region. Their transcriptional start sites should be the same as those that were determined in our 5’ RACE experiments or by previously published primer extension experiments [26]. Additional studies should investigate the possibility of interactions between RpoE and RpoD during the transcription of *luxR*.

Similar to its counterpart *hapR*, *luxR* should also be strictly regulated by a sophisticated network of multiple *cis-* and *trans-*acting factors. Indeed, our EMSA and DNase I footprinting assays provided evidence indicating a complex interplay between RpoE with AphA and LuxR at specific binding sites in the *luxR* promoter region (Fig 6). Two conserved LuxR binding sites [26] were also mapped in the upstream and downstream of the RpoE binding site in P_*luxR*_. Intriguingly, it was also observed that both of the LuxR binding sites, LBSI and LBSII, were involved in *luxR* transcription. The LuxR binding LBSII repressed *luxR* transcription, as previously reported [67], which was expected because there was overlap between the binding sites for RpoE (or potentially RpoD) and LBSII. These results indicated that the binding of RNAP to its binding site was occluded to some extent. When LBSI was absent, the RpoE-mediated activation of *luxR* expression was abolished (Fig 6E), strongly indicating that this *cis*-region might be involved in the attachment of RpoE to its binding site (similar to a UP element) or in subsequent steps during transcription. This result is different from a previous observation [67], and the reason for this discrepancy is unclear. Other factors, such as Fis and HNS, may also be involved in LBSI-mediated RpoE binding or subsequent steps. A previous investigation indicated that LBSI and LBSII showed a low level or a lack of cooperativity with σ^70^-based RNAP [67]. We found that LuxR and RpoE cooperatively interact upon binding to their corresponding binding sites, LBSI and RpoEB, respectively, and that LuxR binding to LBSI facilitated RpoE-P_*luxR*_ binding (Fig 6D).

We were intrigued by the finding that temperature is involved in the stimulation of LuxR production in *V*. *alginolyticus* (Fig 3). Our data demonstrate that RpoE binding to the *luxR* promoter is essential for the temperature-mediated regulation of LuxR. At higher temperatures, *rpoE* expression was increased (S2 Fig) and RpoE was released from membrane-bound RseA-RpoE complexes as a result of the activation of the RIP signaling pathway. This process resulted in the accumulation of cytoplasmic RpoE (Fig 7B), subsequent competition with RpoD for RNAP core enzymes [17], and targeting of the *luxR* promoter, which triggered its expression (Figs 3 and 4E). As discussed in Fig 6E, *cis*- and *trans*-acting elements (e.g., AphA, LuxR and the corresponding binding boxes) might not be involved in the sensing of or interactions with temperature variations, and only RpoE and the RpoE binding box (RpoEB) were essential for the temperature-dependent regulation of *luxR* expression. These results suggest that temperature acts in the upstream of this regulatory pathway. In addition, the temperature-dependent process that regulates *luxR* seems to be independent of QS upstream signals because RpoE was able to direct *luxR* expression in LuxO deletion mutants, and the double deletion of *rpoE* and *luxO* resulted in a similar level of *luxR* expression (Fig 3).

**Fig 9:**
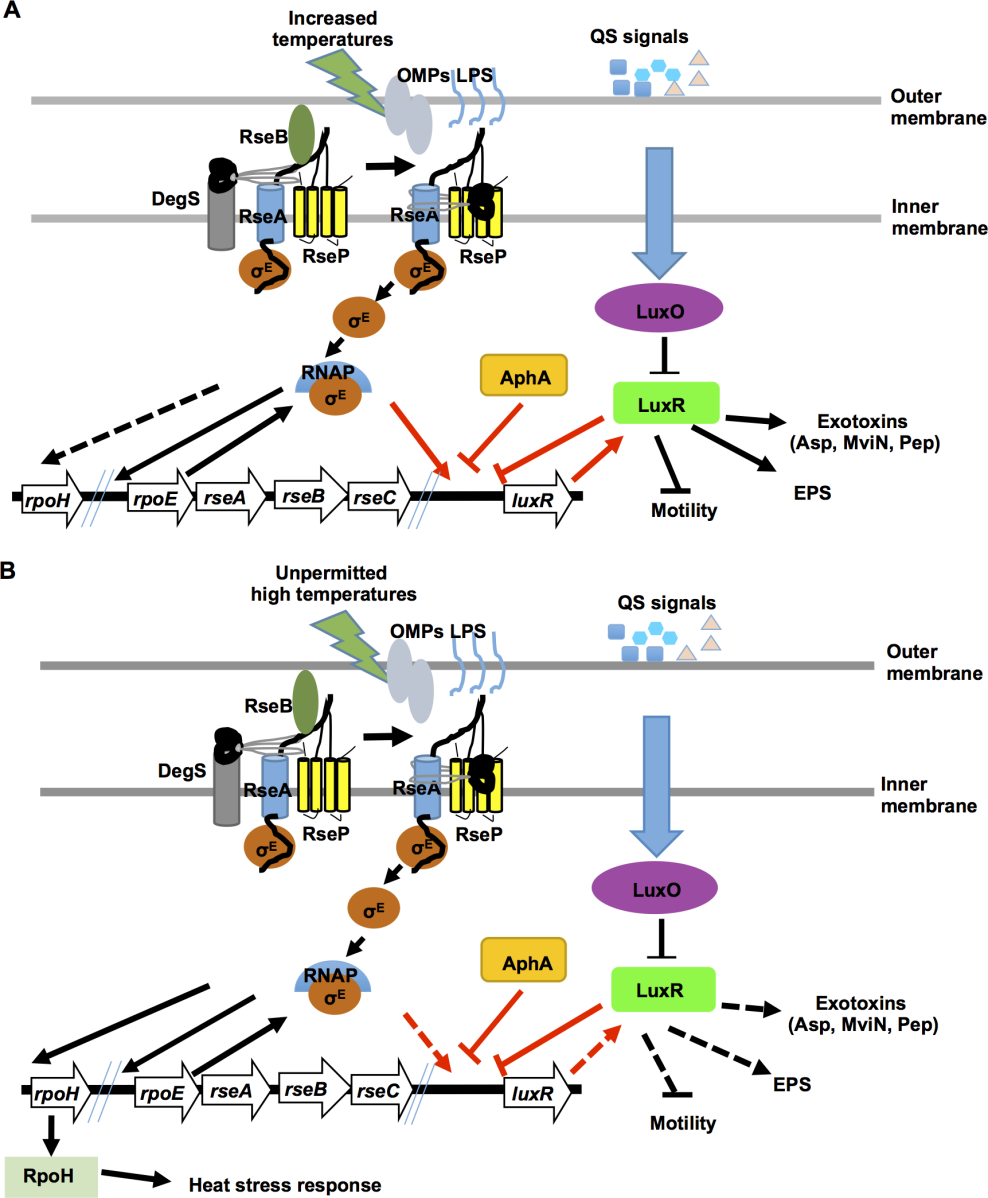
Working model for RpoE switching from QS-controlled virulence to heat stress adaptation at increasing temperatures. (**A**) As temperatures increase (e.g., from 30°C to 37°C), unknown sensor(s) trigger the RIP pathway bind σ^E^ to RseA on the inner membrane. σ^E^ is then incorporated into RNAP to activate (solid lines) the expression of *luxR* and QS. (**B**) When temperatures increase to higher, unpermitted conditions (e.g., 42°C), RIP signaling releases the inner membrane bound σ^E^ to activate the expression of *rpoH* or other factors, which initiates the response to heat stress to improve survival and switch off the expression of LuxR and QS (dashed lines). The *rpoE-resABC* and *luxR* loci are shown. The temperature-dependent σ^E^ switch, which can turn *luxR* expression on/off by interacting with AphA/LuxR, is highlighted with red arrows and bar-ended lines. Factors that may be involved in RIP signaling are shown and discussed in the text.

We propose that the following processes are involved in the cell density- and temperature-dependent transcriptional activation of *luxR* in *V*. *alginolyticus* (Fig 9). At low cell densities, AphA and basal levels of LuxR repress the transcription of *luxR*, while *qrrs* facilitate the degradation of leaked *luxR* mRNA to control its translation. At high cell densities, AphA levels are lower, and the reduction of LuxR binding to LBSI promotes RpoE binding to specific sites. This removes the repression of LuxR binding to LBSII and allows the initiation of the transcription of *luxR* and the production of virulence factors (Fig 9A). In addition, under normal conditions, RpoE is expressed, but it is sequestered by RseA to the inner membrane. As the environmental temperature increases, outer membrane proteins (OMPs) or LPS sensors trigger the activity of DegS and other proteolytic enzymes (i.e., RseP). This activates the RIP pathway and releases RpoE into the cytoplasm. RpoE is then incorporated into the RNAP core enzyme and subsequently activates *luxR* expression and the production of QS-related markers (e.g., the expression of the exotoxins Asp, MviN and Pep) (Fig 9A). When the environmental temperature is increased to dangerously high levels (e.g., 42°C or higher), RpoE is released from the membrane and incorporated into RNAP core enzymes, which favors both the expression of *rpoH* and other factors that are involved in survival responses to heat stress and the dissociation of core enzyme complex binding to *luxR* promoter regions, which minimizes and then switches off the expression of LuxR and QS to save energy that is needed for fitness and survival under these conditions (Fig 9B).

Because the MQSR promoter region is highly conserved across various vibrios, our results may explain why mass mortalities and animal diseases that are caused by pathogenic vibrios are more common in the summer seasons or, as shown in our experiments (Fig 8A), when temperatures are higher. In conclusion, we identified links between temperature and σ^E^- and QS-regulated virulence, and these links suggest that studying mass mortality events in the warmer summer months may facilitate the development of anti-virulence therapies against pathogenic vibrios.

## Materials and Methods

### Bacterial stains, plasmids and culture conditions

The strains and plasmids that were used in this study are listed in S1 Table. The EPGS (*lacZ* ^+^) strain was constructed by integrating a *lacZ* gene after the *glmS* locus with the suicide plasmid pDM4 [68] (S1 Table). Deletion mutant strains or complement strains were constructed as previously described using specific primers and isothermal assembly (S2 Table) [69,70]. Three sequential runs of isolation of Δ*rpoE* mutants were performed. The whole *ompU* gene in the Δ*rpoE* mutants (n = 13) was amplified and sequenced and compared to that of wt to exclude the possibility of suppressor mutation in *ompU* gene. The constructs that were used for the RpoE, AphA, and LuxR expression analyses were constructed in the plasmid pET22b or pET28a, and the *luxR* promoter (P_*luxR*_) activity assays using pDM8 were generated as previously described [68]. *V*. *alginolyticus* strains and derivatives were normally grown at 30°C in Luria-Bertani (LB) medium supplemented with 3% NaCl (LBS). *Escherichia coli* strains were grown at 37°C in LB medium. When appropriate, the medium was supplemented with ampicillin (100 μg/ml), chloramphenicol (25 μg/ml), kanamycin (50 μg/ml), or L-arabinose (0.04%, wt/vol).

### Extracellular protease activity assay and immunoblots of Asp and LuxR

ECP activity was determined using hide powder azure (HPA) (Sigma, St. Louis, Mo, USA) digestion as previously described [69]. Briefly, the strains were grown in LBS medium at 30°C for 9 h. The cell density was measured at 600 nm (OD_600_). The bacterial cultures were centrifuged, and the supernatants were then filtered through 0.22 μm filters (Millipore, Bedford, MA, USA), and 1 ml of the filtered supernatants was mixed with 1 ml of phosphate buffered saline (PBS, pH 7.2) and 10 mg of HPA. This mixture was incubated while shaking at 37°C for 2 h. After stopping the reaction by adding 10% trichloroacetic acid, total protease activity was measured at 600 nm. ECP activity was normalized for each strain by dividing the total activity by OD_600_.

For the Asp and LuxR immunoblotting assays, bacterial cell pellets or concentrated ECP were suspended in 1 volume of PBS to normalize the culture densities based on OD_600_. A 20 μl volume of each normalized sample was loaded onto a 12% denaturing polyacrylamide gel. The proteins were resolved using electrophoresis and then transferred to PVDF membranes (Millipore). The membranes were blocked in 10% skim milk powder solution, incubated with a 1:2,000 dilution of Asp- or LuxR-specific rabbit antibodies (GL Peptide Ltd., Shanghai, China), and then incubated with a 1:2,000 dilution of a horseradish peroxidase-conjugated goat anti-rabbit IgG (Santa Cruz Biotechnology, Santa Cruz, CA, USA). For loading controls, the same blots that were used for the cell lysates were stripped and then incubated with a 1:20,000 dilution of mouse monoclonal anti-RNA polymerase alpha subunit antibodies (RNAP alpha) (4RA2 antibody; Santa Cruz Biotechnology) following by a 1:10,000 dilution of anti-mouse peroxidase-conjugated IgG secondary antibodies (Sigma). Finally, the blots were visualized using ECL reagent (Thermo Fisher Scientific Inc., Waltham, MA USA).

### β-galactosidase activity assay

Bacterial cells were sampled at the indicated time points and then assayed for *β*-galactosidase activity as previously described [68]. Cell pellets were obtained from 200 μl of the cultures at indicated time. The pellets were resuspended in 0.7 ml of PM buffer (60 mM Na_2_HPO_4_, 40 mM NaH_2_PO_4_, 10 mM KCl, and 1 mM MgSO_4_, pH 7.0), and 30 μl of chloroform and 30 μl of 0.1% SDS were added. The solution was then vigorously vortexed to lyse the bacterial cells. To start the reaction, 200 μl of *o*-nitrophenyl-*β*-D-galactoside (4 mg/ml) (Sigma) was added to the solution. When the suspension started to turn yellow, the reaction was stopped by adding 0.4 ml of 1 M Na_2_CO_3_. The mixture was centrifuged, and the supernatant was used to measure the absorbance at 420 nm. *β*-galactosidase activity was calculated according to following formula: A_420_ × 1,000 × min^-1^× ml^-1^× A_600_^-1^.

### Total RNA extraction

Bacteria were incubated overnight and then diluted 1:100 in LBS. They were then grown at 30°C, 37°C or 42°C and harvested after 9 h. Total RNA was isolated using an RNA extraction kit (Tiangen, Beijing, China). The RNA samples were digested with DNase I (Promega, Madison, WI, USA) to eliminate genomic DNA contamination. Before reverse transcription, regular PCR was routinely performed using the isolated RNA sample as a template to confirm that there was no DNA contamination.

### Quantitative real-time reverse transcription PCR (qRT-PCR)

Equal amounts of RNA (1 μg) were used to generate complementary DNA (Toyobo, Tsuruga, Japan) using random primers. Three independent qRT-PCR experiments were performed, and each was run in triplicate. The specific primer pairs that were used are shown in S2 Table. Reactions were run on an Applied Biosystems 7500 Real Time System (Applied Biosystems), and transcript levels were normalized to 16 sRNA in each sample using the ΔΔ*C*_T_ method.

### 5’-RACE (Rapid Amplification of cDNA Ends)

We performed 5’-RACE experiments as previously described [71]. Six micrograms of total RNA was extracted from cultures of wt and Δ*rpoE* strains and subjected to dephosphorylation using Tobacco Acid Pyrophosphatase (TAP) (Epicentre) for 60 min at 37°C. The RNA oligo linker was ligated to total RNA using T4 RNA ligase (New England Biolabs, Beverly, MA, USA) according to the manufacturer’s instructions. cDNA was synthesized using AMV RT (TaKaRa) according to the manufacturer’s instructions using a random primer. The first-round PCR amplification was performed using a RACE-adapter and the primer *luxR*-RACE (S2 Table), and the second-round PCR amplification was performed using RACE-adapter-nested primers and *luxR*-RACE-nested primers (S2 Table). The single resulting band was extracted, sub-cloned and sequenced.

### *In vitro* transcription and RT-PCR

Linear DNA templates containing the *luxR* promoter region and the first 112 bp of the *luxR* ORF were generated using PCR. A positive control DNA template containing the *rpoE* promoter and the first 121 bp of the *rpoE* gene was also amplified. *In vitro* transcription was performed as previously described, with modifications [72], according to the manufacturer’s protocols. Briefly, 1 unit of *E*. *coli* RNA polymerase core enzyme (NEB) was incubated with 0.3 μg of template DNA and RpoE protein in transcription buffer (40 mM Tris-HCl, pH 7.5, 150 mM NaCl, 10 mM MgCl_2_, 1 mM DTT and 0.01% Triton X-100) at 37°C for 40 min to allow open complexes to form. LuxR was added to the mixture as indicated. RNA synthesis was initiated by adding 2 μl of NTP mixture, which contained 0.5 mM ATP, CTP, GTP and UTP (NEB). After 15 min of incubation at 37°C, the reactions were stopped by extracting the RNA using phenol and purifying the RNA using ethanol precipitation. The RNA was eluted using 15 μl of water and stored at -80°C. The purified RNA was treated with DNase I (Promega), and 8.0 μl of the RNA was then digested in a final reaction volume of 10 μl. To perform RT with a Toyobo transcript kit, 7 μl of the digestion reaction and 0.5μl of *luxR*rev1 primer (S2 Table) were used in a final reaction volume of 10 μl. After the reaction, 1 μl of the RT mix was transferred to PCR tubes containing 19 μl aliquots of PCR mix. PCR amplification was performed using the *luxR*rev1 and *luxR*for1 primers (S2 Table) and resolved on 1.5% agarose gels using electrophoresis.

### Electrophoretic mobility shift and DNase I footprinting assays

For the electrophoretic mobility shift assays (EMSA), purified ^6^His-tagged RpoE, AphA, or LuxR was incubated with different Cy5-labeled DNA probes (S2 Table) in 20 μl of loading buffer (10 mM NaCl, 0.1 mM DTT, 0.1 mM EDTA, 10 mM Tris, pH 7.4). After the mixture was incubated at 25°C for 30 min, the samples were resolved using 6% polyacrylamide gel electrophoresis in 0.5× TBE (Tris/Boric Acid/EDTA) buffer on ice at 100 V for 120 min. Then, the gels were scanned using a Typhoon FLA 9500 (GE healthcare, Uppsala, Sweden).

Dye primer-based DNase I footprinting assays were performed as previously described [73]. Briefly, the promoter region of *luxR* was PCR-amplified using *pfu* DNA polymerase and M13F-47 and M13R-48 primers, which carry 6-FAM at the 5’ end (S2 Table). The FAM-labeled probes were purified using a Wizard SV Gel and PCR Clean-Up System (Promega) and quantified using a NanoDrop 2000C (Thermo Fisher Inc.). For each assay, 400 ng of the probes were incubated with different amounts of RpoE in a total volume of 40 μl. After the mixture was incubated for 30 min at 25°C, a 10 μl solution containing approximately 0.015 units of DNase I (Promega) and 100 nmol of freshly prepared CaCl_2_ was added. The mixture was then incubate for 1 min at 25°C. The reaction was stopped by adding 140 μl of DNase I stop solution (200 mM unbuffered sodium acetate, 30 mM EDTA and 0.15% SDS). The samples were first extracted using phenol/chloroform and then precipitated using ethanol, and the pellets were dissolved in 10 μl of MiniQ water. Approximately 2 μl of digested DNA was added to 7.9 μl of HiDi formamide (Applied Biosystems) and 0.1 μl of GeneScan-500 LIZ size standards (Applied Biosystems). The samples were analyzed using a 3730 DNA Analyzer with a G5 dye set that was run on an altered default genotyping module that increased the injection time to 30 s and the injection voltage to 3 kV. The results were analyzed using GeneMapper 4.0 (Applied Biosystems).

### Chromatin immunoprecipitation quantitative PCR (ChIP-qPCR)

ChIP-qPCR was performed as previously described [74]. Cultures of wt and Δ*rpoE* strains were cultured overnight and then diluted 1:100 in 50 ml of fresh LBS medium. After 9 h of growth while shaking at different temperatures (22°C, 30°C, 37°C, or 42°C), the bacteria were treated with 1% formaldehyde at room temperature for 10 min to perform *in vivo* cross-linking of protein-DNA complexes. The reaction was stopped by adding 125 mM glycine. The bacteria were then washed twice with cold PBS and resuspended in 5 ml of SDS lysis buffer (50 mM HEPES-KOH pH 7.5, 0.1% Sodium Deoxycholate, 150 mM NaCl, 0.1% SDS, 1 mM EDTA pH 8, 1% Triton X-100, and a protease inhibitor cocktail). Then, the bacteria were sonicated, and the DNA was fragmented to 100–500 bp at 200 W. Insoluble cellular debris was removed via centrifugation, and the supernatant was used as the input sample in the following IP experiments. Both the input and the IP samples were washed with 50 μl of protein G beads for 1 h, and then the IP samples were incubated overnight with 5 μl of RpoE-specific antibodies (1RE53 antibody, Santa Cruz). They were then incubated with 50 μl of protein G beads for 1 h. The beads were washed twice using a low salt wash buffer (10 mM Tris-HCl pH 8, 150 mM NaCl, 0.1% SDS, 1 mM EDTA pH 8, and 1% Triton X-100), twice with 1 ml of a high salt wash buffer (10 mM Tris-HCl pH 8, 500 mM NaCl, 0.1% SDS, 1 mM EDTA pH 8, and 1% Triton X-100), twice with 1 ml of a LiCl wash buffer (10 mM Tris-HCl pH 8, 25 0 mM LiCl, 1 mM EDTA pH 8, 0.5% Triton X-100, and 0.5% Sodium Deoxycholate), and twice with 1 ml of TE buffer. The beads were resuspended in 200 μl of elution buffer (50 mM Tris HCl pH 8, 10 mM EDTA, and 1% SDS), incubated at 65°C for 2 h, and then centrifuged at 5000 *g* for 1 min. The supernatants containing the immunoprecipitated DNA were collected, and 8 μl of 5 M NaCl was added to all of the tubes (IPs and Inputs). The tubes were then incubated at 65°C overnight to reverse the DNA-protein crosslinks. Then, 1 μl of RNase A was added, and the solutions were incubated for 30 minutes at 37°C. A 4 μl volume of 0.5 M EDTA, 8 μl of 1 M Tris-HCl and 1 μl of proteinase K were added to each tube, and the tubes were incubated at 45°C for 2 h. The DNA was purified using phenol-chloroform and amplified using qPCR with the primers listed in S2 Table.

The enriched DNA targets were calculated using the following ΔΔ*C*_T_ method. For each DNA target, Δ*C*_T_ of the Input fraction and IP fraction was calculated in both the wt and Δ*rpoE* samples. Each value was then divided by the corresponding Δ*C*_T_ that was obtained for the non-specific *gyrB* intragenic region in the strains. Then, the enrichment ratio was calculated from the ΔΔ*C*_T_ value in wt strain divided by that of Δ*rpoE* strain.

### Fluorescence assay

Cultures were cultured overnight and then diluted 1:100 in fresh LBS medium containing 0.004% arabinose or no arabinose. After 9 h of growth while shaking at different temperatures (22°C, 30°C, 37°C, or 42°C), the bacterial cells were washed twice with PBS. Then, YFP, CFP, and mCherry fluorescence was respectively measured using a florescence plate reader (Biotek, Winooski, VT, USA) in samples with or without arabinose induction.

### RpoE localization assay

The plasmid pBAD33::*rpoE* was transformed into wt, Δ*degS* and Δ*rseA* ^20-39^ cultures. The cultures were then incubated overnight and diluted 1:100 in fresh LBS medium containing 0.004% arabinose. After 9 h of growth while shaking at different temperatures (22°C, 30°C, 37°C, or 42°C), the bacterial cells were washed twice with cold PBS and resuspended in 15 ml of PBS. The bacterial cells were then high-pressure crushed, and the suspensions were first centrifuged at 3,000 g for 10 min to remove the intact cells, and then at 100,000 g for 1 h. The precipitated pellets contained membrane parts while the supernatant pellets contained cytoplasmic proteins. Western blot analysis was performed to detect the amount of RpoE in the membrane and cytoplasmic pellets.

### Survival assays

Cultures of *V*. *alginolyticus* were incubated overnight in LBS at 30°C with aeration. The cultures were diluted in the same medium to an OD_600_ of 0.001 and then incubated until they reached an OD_600_ of 1.0 (1 ×10^9^ cells/ml). Osmotic challenge (30% sucrose), ethanol challenge (10% ethanol), and oxidative challenge (10 mM H_2_O_2_) were applied to the cultures in LBS medium at 30°C. Heat challenge was performed in pre-warmed (42°C) LBS medium. At the indicated time points, an aliquot was taken from each culture to determine the survival rate using plate counts after appropriate dilutions. Every experiment was repeated at least three times, and the values are shown as the means of triplicate samples.

### Infection of fish

Wt and Δ*rpoE* bacteria were incubated at 30°C overnight and then inoculated into fresh LBS medium. The cultures were grown for 9 h. All of the strains were harvested and then serially diluted with PBS. Zebrafish weighing approximately 0.25 g were infected with the strains at doses ranging from 10^4^ to 10^7^ CFU/fish via intramuscular (i.m.) injection according to a previously published description [75]. Zebrafish were anesthetized with tricaine methanesulfonate (MS-222) (Sigma-Aldrich) at a concentration of 80 mg/l. Thirty fish were infected with each dilution, and three parallel experiments were performed. Then, the fish were cultured at different temperature, and death that was caused by vibriosis was observed and confirmed by isolating *V*. *alginolyticus* strains from the dead fish. LD_50_ values were calculated according to a previously described method [75].

### Competitive index assays

The competitive indexes (CI) of the wt and Δ*rpoE* strains were determined using a wt strain that harbored a *lacZ* gene behind the *glmS* locus (S1 Table). The infection dose was 10^5^ CFU/fish. Seven zebrafish were sacrificed at 24 h post-infection and then ground and plated in LBS containing X-Gal and Amp. The plates were cultured overnight at 30°C, and the wt strain was differentiated from the mutant strains based upon the production of blue and white colonies (wt strains were blue). The ratios of Δ*rpoE* counts to the wt counts were used to determine the CI.

### Ethics statement

All animal experiments presented in this study was approved by the Animal Care Committee of the East China University of Science and Technology (2006272). The Experimental Animal Care and Use Guidelines from Ministry of Science and Technology of China (MOST-2011-02) was strictly adhered.

## Supporting Information

S1 Fig The growth and membrane integrity of wt, Δ*rpoE* and *rpoE* ^+^ complement strains in LBS medium at various temperatures. **(A)** Growth curves of wt, Δ*rpoE* and *rpoE* ^+^ complement strains in LBS medium at different temperatures. The cultures were sampled at various time points and plate-counted after series dilutions with fresh LBS medium. **(B)** The wt, Δ*rpoE* and *rpoE* ^+^ strains were cultured in LBS for 9 h at 30°C and 42°C (left), and western blot analysis was performed to determine RNAP in the pellet and supernatant of wt, Δ*rpoE* and *rpoE* ^+^ (right). The analysis was performed using concentrated supernatants (S) and cellular pellets (C) with RNAP antibody. All the samples were normalized by OD_600_ values. The numbers under each lane correspond to densitometry measurements.
(TIFF)

S2 Fig Temperature responsive *rpoE* and *rpoS* expression in EPGS. qRT-PCR was carried out with RNA samples extracted from 9 h cultures at various temperatures as described in Materials and Methods. The results are normalized by the 16S rRNA gene using the ΔΔ*C*_T_ method and the difference relative to the levels cultured in 22°C are shown. * *P* <0.05, ** *P*<0.01, ** *P<0.001, *t-*test.
(TIFF)

S3 Fig Absence of the specific binding site abolished the capability of RpoE and LuxR binding to *luxR* promoter. **(A)** EMSAs of purified RpoE binding to the *luxR* promoter region (P_*luxR*_) (lanes 1–2) and P_*luxR*_ with the specific RpoE binding site deleted (P_*luxR*_ΔRpoEB) (lanes 3–8). (**B)** EMSAs of purified LuxR binding to the P_*luxR*_ with the specific LuxR binding site I and II deleted (P_*luxR*_ΔLBSI/II). The amounts of RpoE and LuxR protein used were as indicated and 20 ng of each Cy5-labelled probe as well as non-specific competitor DNA (poly(dI:dC)) were added to the EMSA reactions.
(TIFF)

S4 Fig EMSAs of purified RpoE binding to the *luxR*, *rpoH*, and *rpoE* promoter regions (P_*luxR*_, P_*rpoH*_, and P_*rpoE*_) at various temperatures. The amounts of RpoE protein used were as indicated and 20 ng of each Cy5-labelled probe as well as non-specific competitor DNA (polydI:dC) were added to the EMSA reactions at indicated temperatures. All the EMSA reactions were performed at least 3 times and the representative one result was shown.
(TIFF)

S5 Fig Interaction of LuxR and AphA to RpoE in regulation of *luxR* expression. (**A**) P_*luxR*_ activities in wt, Δ*luxR*, and Δ*aphA* strains. The wt, Δ*luxR*, and Δ*aphA* strains carrying the P_*luxR*_-*lacZ* reporter plasmid were cultured in LBS medium and assayed for *β*-galactosidase activity. Results were presented as mean ± S.D. (*n* = 3). (**B-C**) EMSA assays of various AphA, LuxR, and RpoE mixtures binding to *luxR* promoter region. Arrows indicated the specific bands of corresponding proteins shifted with the *luxR* DNA.
(TIFF)

S6 Fig Genetic and interaction analysis of *rpoE* and *rseA* from *V*. *alginolyticus*. (**A**) *rpoE-rseABC* locus is highly conserved among *V*. *alginolyticus*, other vibrios, and *E*. *coli*. (**B**) Diagram of established interaction of RpoE and RseA in *E*. *coli*. **(C-D)**. Bacterial two-hybrid system to assay the interaction of RpoE and RseA in *V*. *alginolyticus* as qualitatively and quantitatively determined on X-gal plate (**C**) and *β*-galactosidase activity assays (**D**).
(TIFF)

S7 Fig *rpoE* is involved in various stress responses and motilities in *V*. *alginolyticus*. (**A-D**) Wild type, Δ*rpoE*, *rpoE* ^+^ exposed to 30% sucrose, 10% ethanol, heat (42°C), and 10 mmol/L H_2_O_2_. (**E-F**) The growth curve of wt, Δ*rpoE*, *rpoE* ^+^ at 0.5% NaCl and 7% NaCl in LBS medium. All cultures were grown in triplicate, and each experiment was performed at least three times. The viable plate count was carried out at indicated time to determine the survival rate. 100% survival corresponds to the viable cell count determined just prior to exposure to the indicated stress. Error bars indicate the standard deviation for three triplicate samples. (**G-H**) Swarming motility assays of WT, Δ*rpoE* and *rpoE*+ on LBS agar plates containing 1.5% agar (G), and swimming motility assays on LBS agar plates containing 0.3% agar (H)
(TIFF)

S8 Fig *rpoE* binding box is ubiquitously present in MQSR promoter regions in other *Vibrios*. (**A**) Gene alignment of MQSR promoter regions in vibrios. The boxes indicated the putative -10 and -35 regions for RpoE binding. (**B**) EMSA showing RpoE from *V*. *alginolyticus* binding to the MQSR promoter region of other vibrios in *Harveyi* clade.
(TIFF)

S1 Table Bacterial strains and plasmids used in this study. (DOCX)

S2 Table Primers used in this study. (DOCX)
