# Supplementary figures and images for: A σE-Mediated Temperature Gauge Controls a Switch from LuxR-Mediated Virulence Gene Expression to Thermal Stress Adaptation in Vibrio alginolyticus
Source: PLoS Pathog. 2016 Jun 2;12(6):e1005645. doi: 10.1371/journal.ppat.1005645 (PMC4890791; doi:10.1371/journal.ppat.1005645)

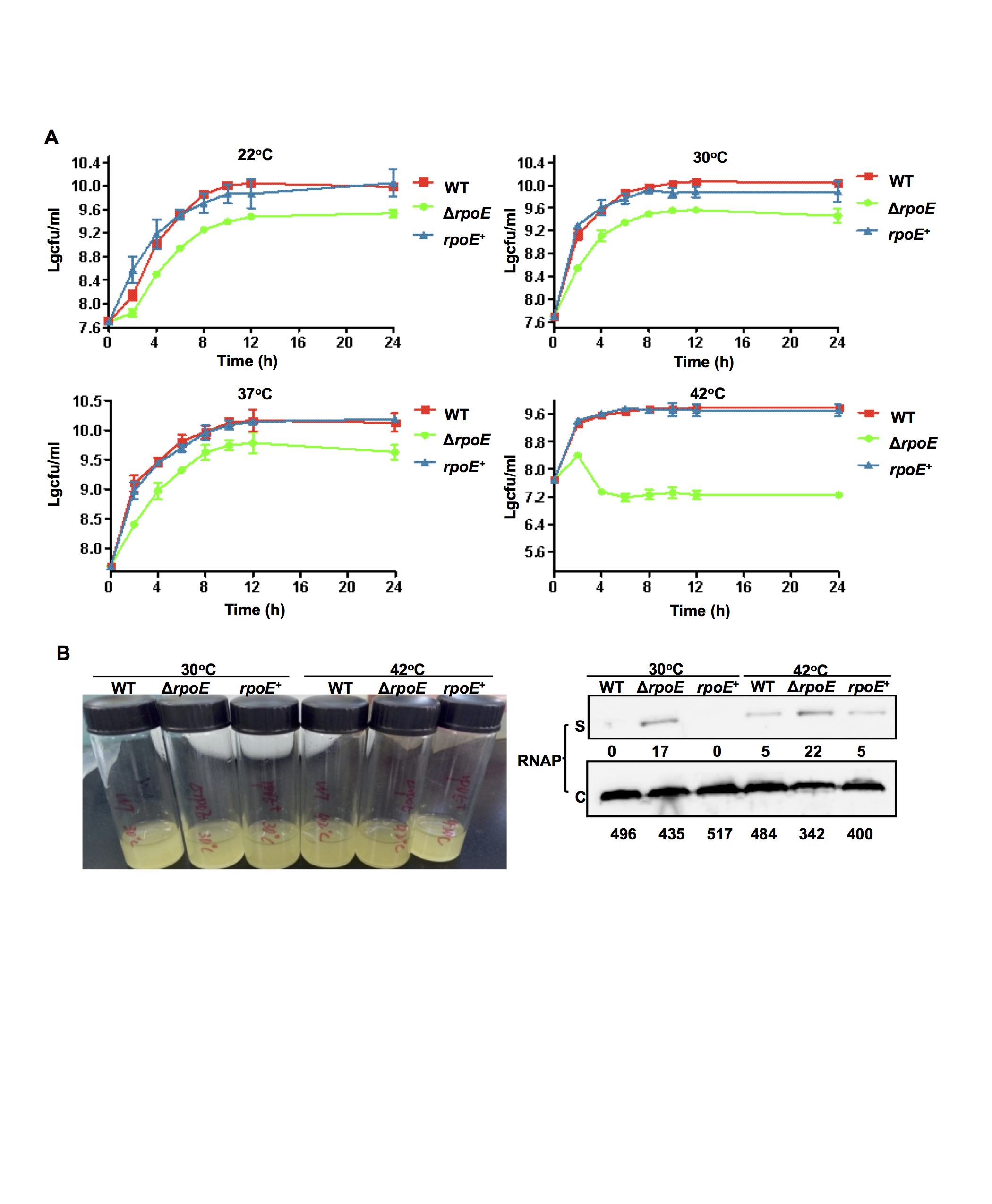

Supplement: S1 Fig — (A) Growth curves of wt, ΔrpoE and rpoE + complement strains in LBS medium at different temperatures. The cultures were sampled at various time points and plate-counted after series dilutions with fresh LBS medium. (B) The wt, ΔrpoE and rpoE + strains were cultured in LBS for 9 h at 30°C and 42°C (left), and western blot analysis was performed to determine RNAP in the pellet and supernatant of wt, ΔrpoE and rpoE + (right). The analysis was performed using concentrated supernatants (S) and cellular pellets (C) with RNAP antibody. All the samples were normalized by OD600 values. The numbers under each lane correspond to densitometry measurements. (TIFF) [file ppat.1005645.s001.tiff]

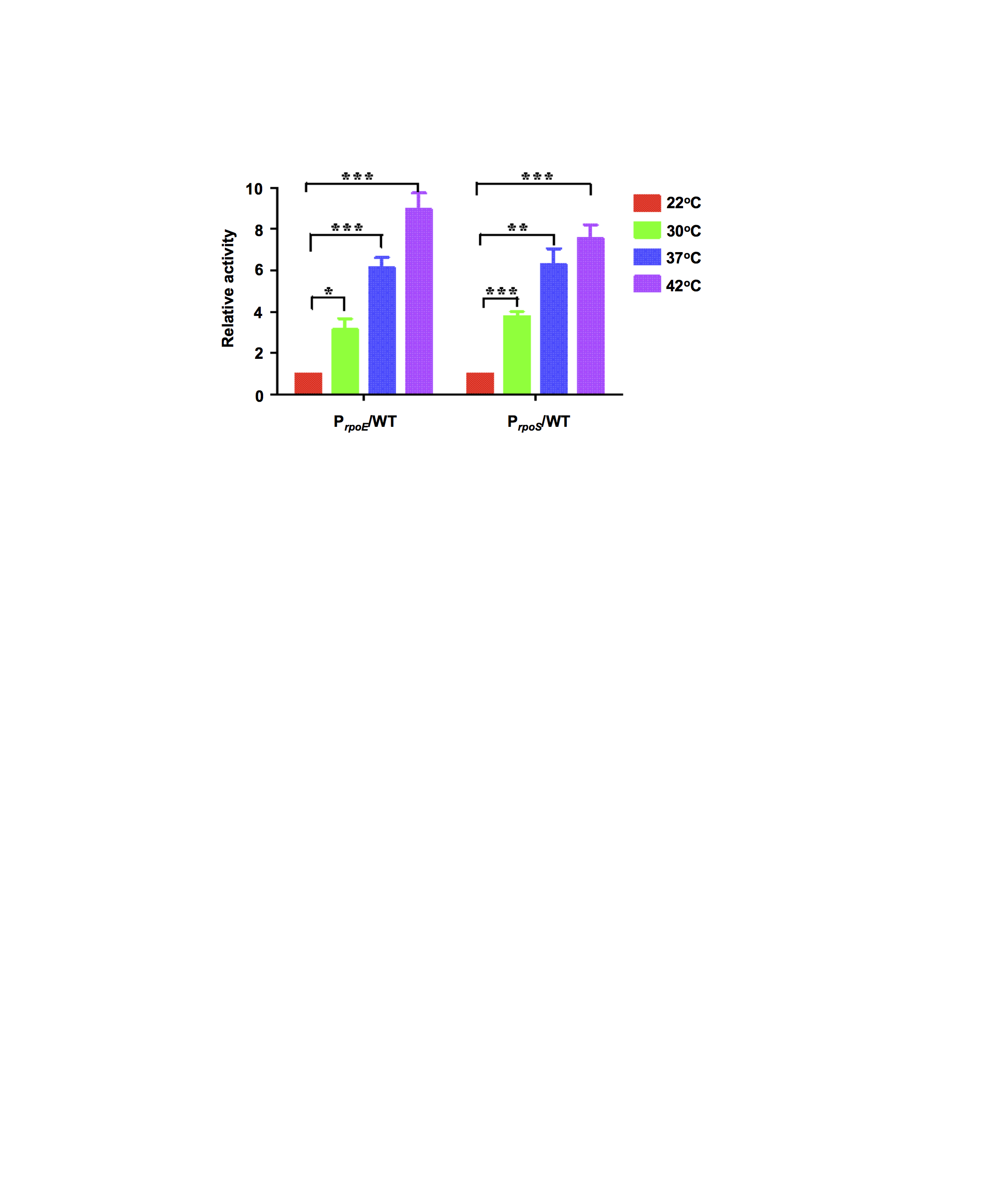

Supplement: S2 Fig — qRT-PCR was carried out with RNA samples extracted from 9 h cultures at various temperatures as described in Materials and Methods. The results are normalized by the 16S rRNA gene using the ΔΔC T method and the difference relative to the levels cultured in 22°C are shown. * P <0.05, ** P<0.01, ** *P<0.001, t-test. (TIFF) [file ppat.1005645.s002.tiff]

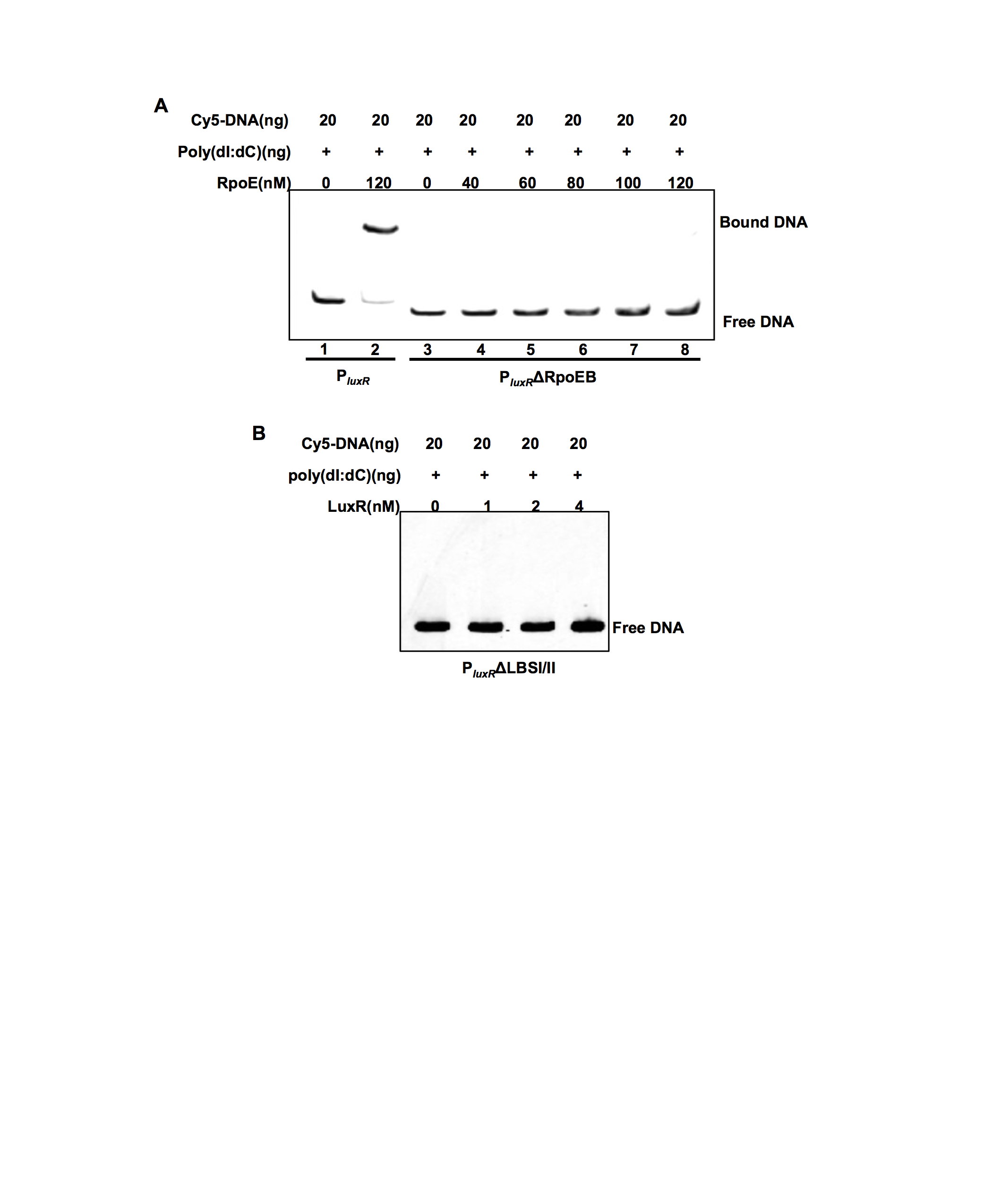

Supplement: S3 Fig — (A) EMSAs of purified RpoE binding to the luxR promoter region (PluxR) (lanes 1–2) and PluxR with the specific RpoE binding site deleted (PluxRΔRpoEB) (lanes 3–8). (B) EMSAs of purified LuxR binding to the PluxR with the specific LuxR binding site I and II deleted (PluxRΔLBSI/II). The amounts of RpoE and LuxR protein used were as indicated and 20 ng of each Cy5-labelled probe as well as non-specific competitor DNA (poly(dI:dC)) were added to the EMSA reactions. (TIFF) [file ppat.1005645.s003.tiff]

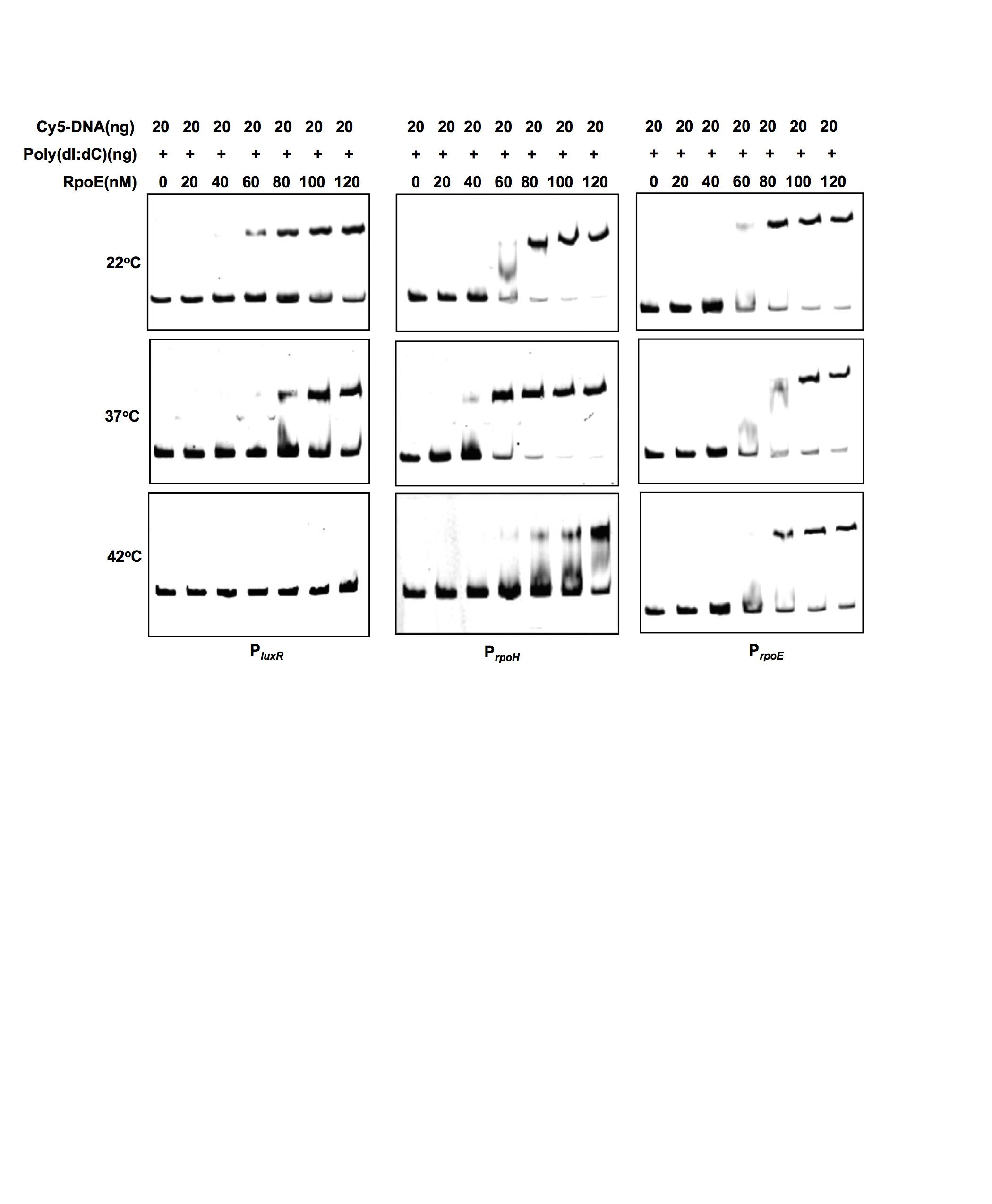

Supplement: S4 Fig — The amounts of RpoE protein used were as indicated and 20 ng of each Cy5-labelled probe as well as non-specific competitor DNA (polydI:dC) were added to the EMSA reactions at indicated temperatures. All the EMSA reactions were performed at least 3 times and the representative one result was shown. (TIFF) [file ppat.1005645.s004.tiff]

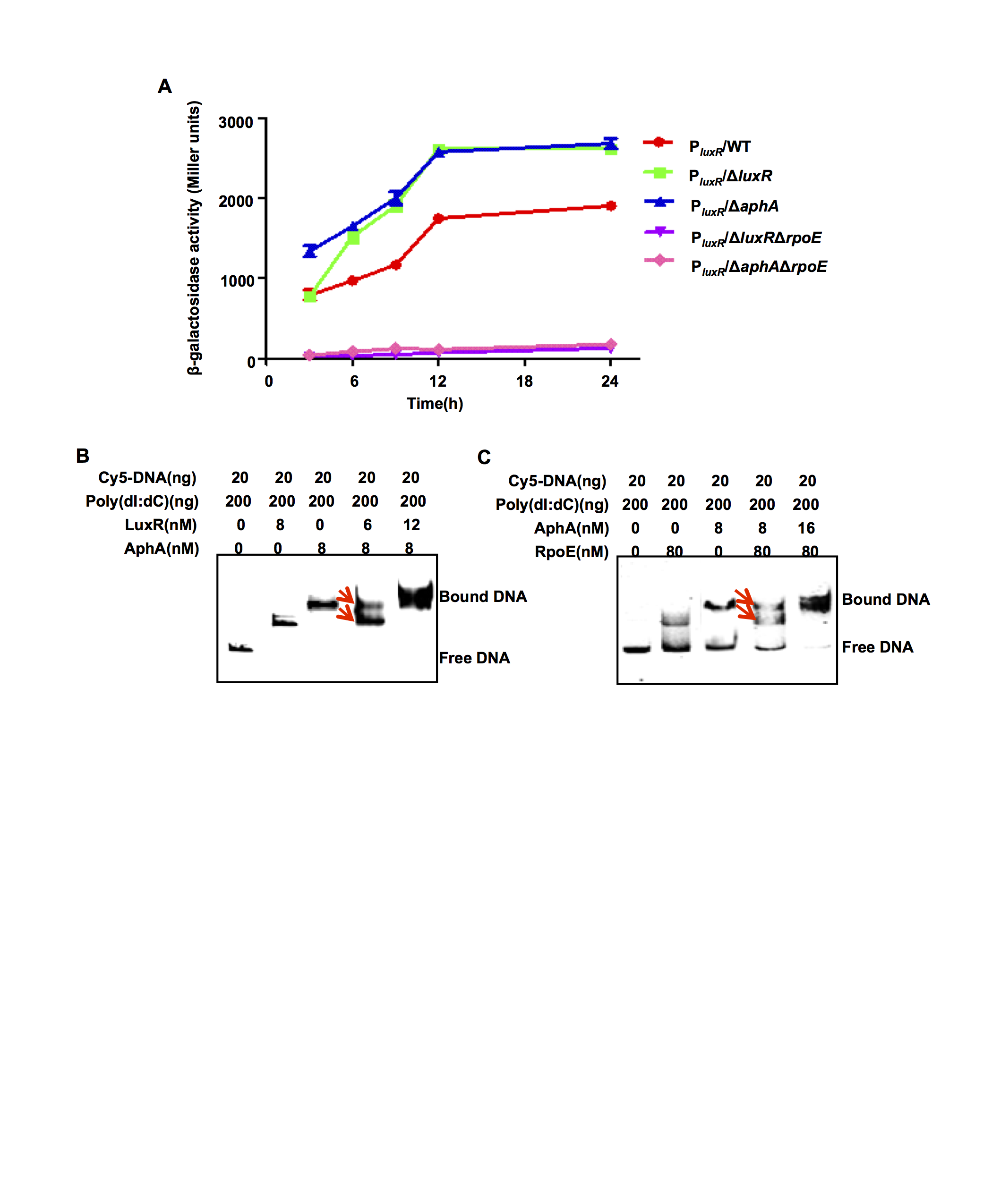

Supplement: S5 Fig — (A) PluxR activities in wt, ΔluxR, and ΔaphA strains. The wt, ΔluxR, and ΔaphA strains carrying the PluxR-lacZ reporter plasmid were cultured in LBS medium and assayed for β-galactosidase activity. Results were presented as mean ± S.D. (n = 3). (B-C) EMSA assays of various AphA, LuxR, and RpoE mixtures binding to luxR promoter region. Arrows indicated the specific bands of corresponding proteins shifted with the luxR DNA. (TIFF) [file ppat.1005645.s005.tiff]

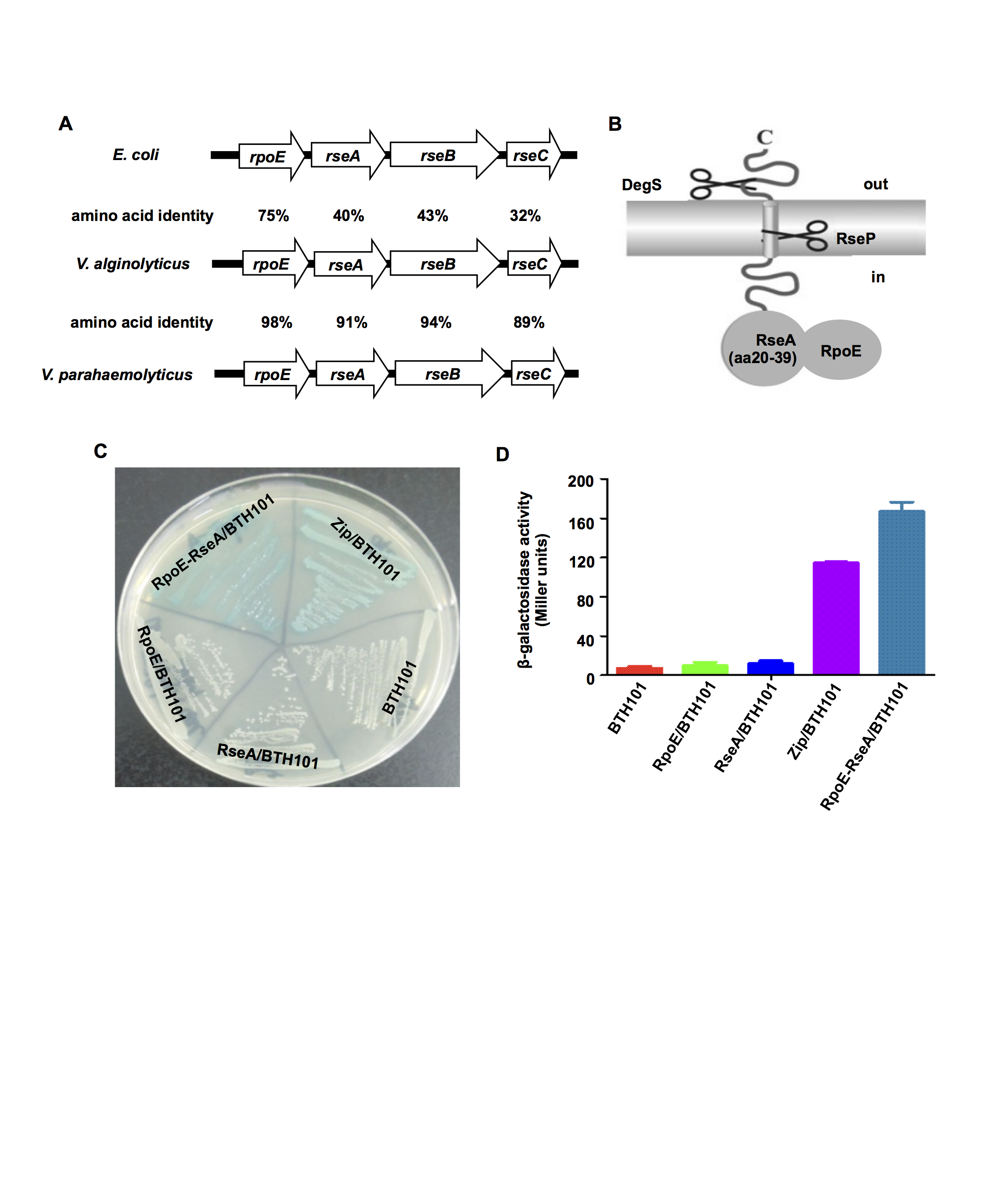

Supplement: S6 Fig — (A) rpoE-rseABC locus is highly conserved among V. alginolyticus, other vibrios, and E. coli. (B) Diagram of established interaction of RpoE and RseA in E. coli. (C-D). Bacterial two-hybrid system to assay the interaction of RpoE and RseA in V. alginolyticus as qualitatively and quantitatively determined on X-gal plate (C) and β-galactosidase activity assays (D). (TIFF) [file ppat.1005645.s006.tiff]

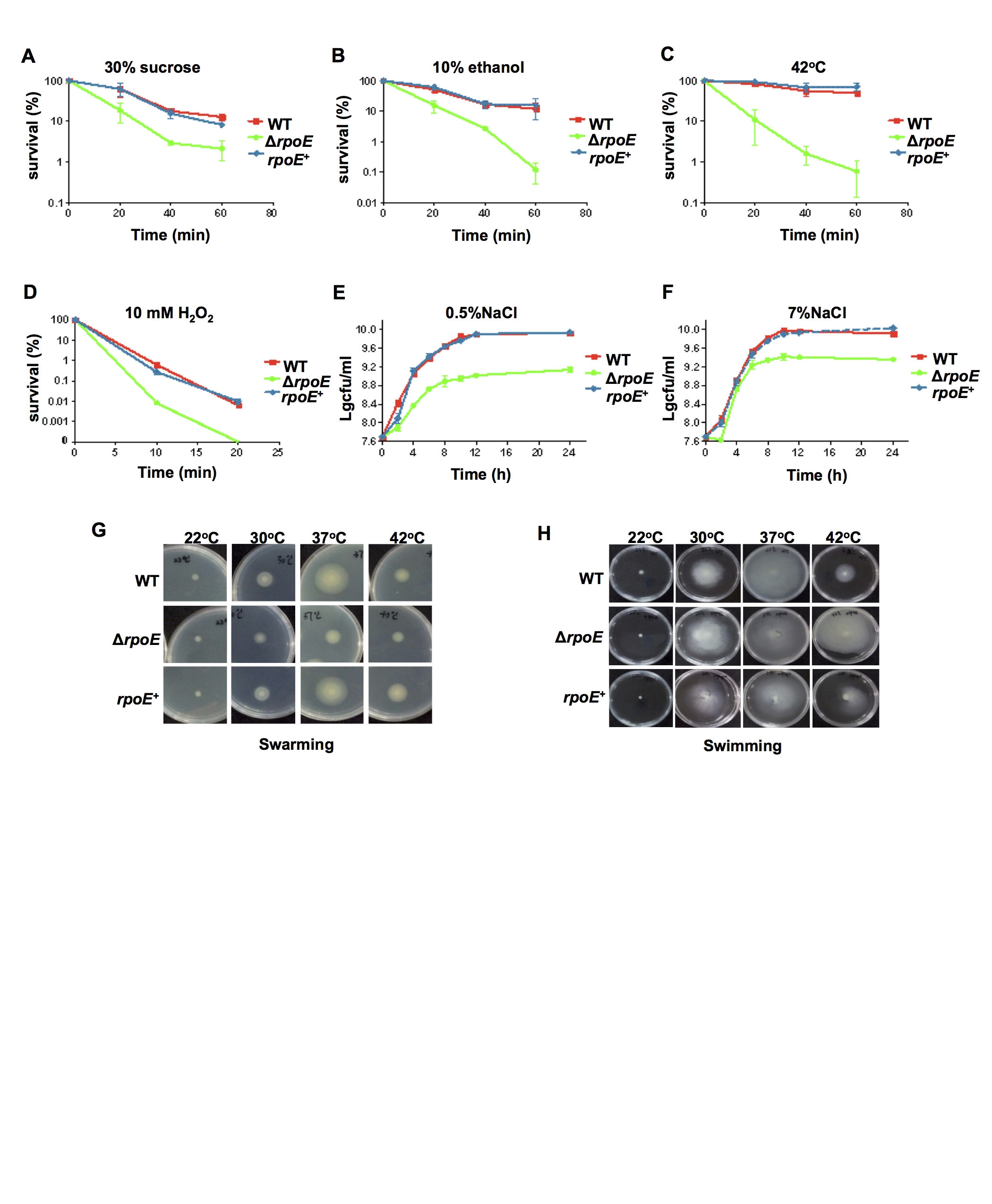

Supplement: S7 Fig — (A-D) Wild type, ΔrpoE, rpoE + exposed to 30% sucrose, 10% ethanol, heat (42°C), and 10 mmol/L H2O2. (E-F) The growth curve of wt, ΔrpoE, rpoE + at 0.5% NaCl and 7% NaCl in LBS medium. All cultures were grown in triplicate, and each experiment was performed at least three times. The viable plate count was carried out at indicated time to determine the survival rate. 100% survival corresponds to the viable cell count determined just prior to exposure to the indicated stress. Error bars indicate the standard deviation for three triplicate samples. (G-H) Swarming motility assays of WT, ΔrpoE and rpoE+ on LBS agar plates containing 1.5% agar (G), and swimming motility assays on LBS agar plates containing 0.3% agar (H) (TIFF) [file ppat.1005645.s007.tiff]

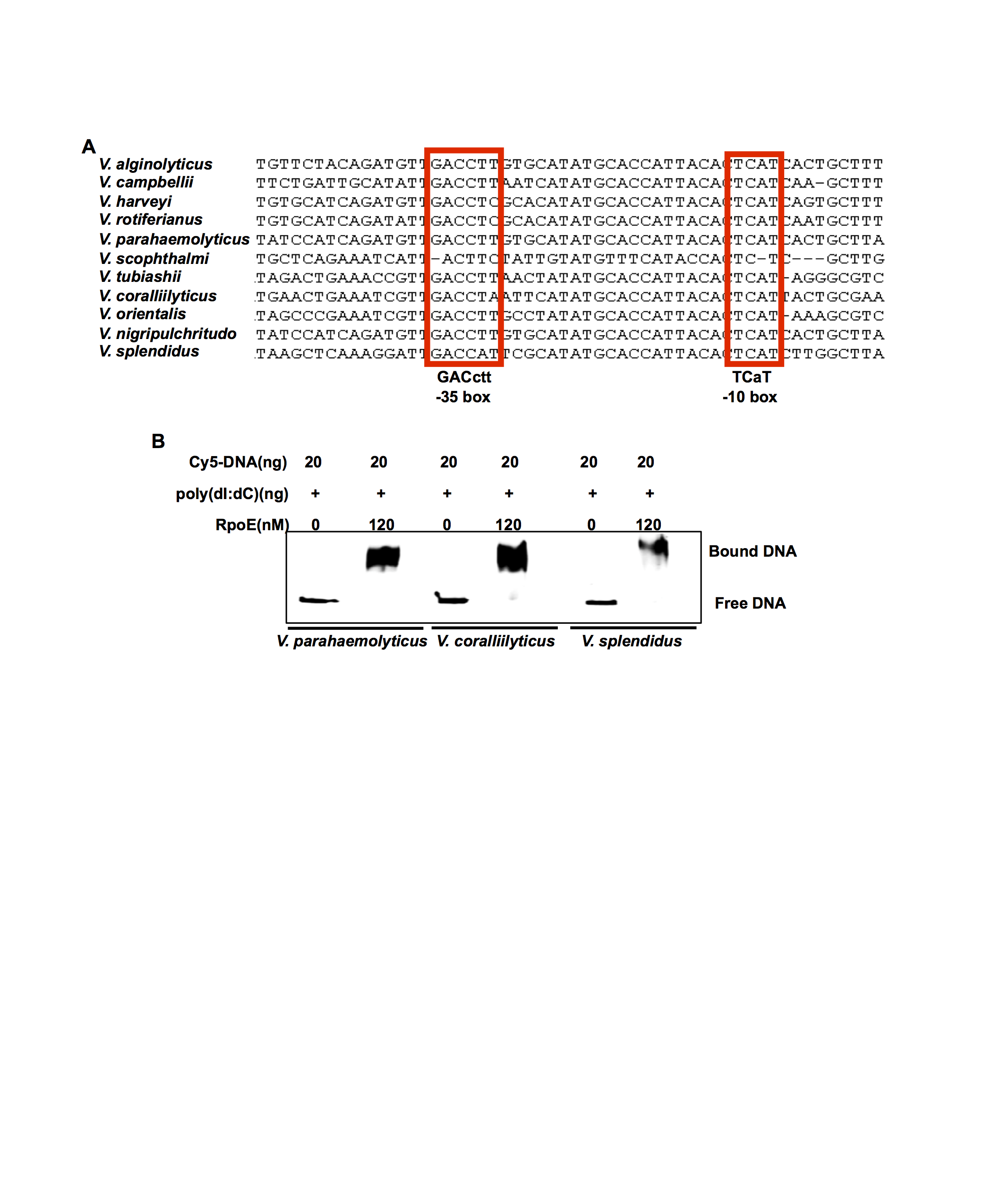

Supplement: S8 Fig — (A) Gene alignment of MQSR promoter regions in vibrios. The boxes indicated the putative -10 and -35 regions for RpoE binding. (B) EMSA showing RpoE from V. alginolyticus binding to the MQSR promoter region of other vibrios in Harveyi clade. (TIFF) [file ppat.1005645.s008.tiff]
