# Supplementary material for: A σE-Mediated Temperature Gauge Controls a Switch from LuxR-Mediated Virulence Gene Expression to Thermal Stress Adaptation in Vibrio alginolyticus
Source: PLoS Pathog. 2016 Jun 2;12(6):e1005645. doi: 10.1371/journal.ppat.1005645 (PMC4890791; doi:10.1371/journal.ppat.1005645)
Supplement: S1 Table — (DOCX) [file ppat.1005645.s009.docx]

**S1 Table. Bacterial strains and plasmids used in this study**

| **Number** | **Strain or plasmid** | **Relevant characteristics** | **Reference** |
| --- | --- | --- | --- |
|  | ***E. coli*** |  |  |
| TS001 | DH5α λ*pir* | Host for π requiring plasmids | Labortaory collection |
| TS002 | SM10 λ*pir* | Host for π requiring plasmids, conjugal donor | (Liang et al. 2003) |
| TS002 | BL21(DE3) | Host strain for protein expression | Novagen |
| TS003 | BTH101 | Reporter strain for Bacterial Adenylate Cyclase Two-Hybrid System | Euromedex |
|  | ***V. alginolyticus*** |  |  |
| AHS001 | EPGS | Wild type, isolated from the aquiculture farm of the South China Sea with CCTCC No. AB 209306. Amp^r^ | Labortaory collection |
| GD001 | wt-*lacZ*^+^ | EPGS, *lacZ* inserted behind *glmS*; Amp^r^ | This study |
| RHP001 | *asp*^-^ | EPGS, disrupted mutant in *asp*; Amp^r^, Cm^r^ | (Rui et al., 2009) |
| RHP002 | Δ*luxR* | EPGS, in-frame deletion in *luxR*, Amp^r^ | (Rui et al., 2009) |
| GD002 | Δ*rpoE* | EPGS, in-frame deletion in *rpoE*, Amp^r^ | This study |
| LH001 | Δ*rpoS* | EPGS, in-frame deletion in *rpoS*, Amp^r^ | (Liu et al., 2011) |
| GD003 | *rpoE*^+^ | Δ*rpoE* complemented strain, Amp^r^, Cm^r^ | This study |
| LH002 | Δ*luxO* | EPGS, in-frame deletion in *luxO*, Amp^r^ | (Liu et al., 2011) |
| GD004 | Δ*rpoE*Δ*luxO* | EPGS, in-frame deletion in *rpoE* and *luxO*, Amp^r^ | This study |
| GD005 | pBAD33::*rpoE*/  Δ*rpoE*Δ*luxO* | Δ*rpoE*Δ*luxO*, pBAD33 expressing the *rpoE* gene, Amp^r^, Cm^r^ | This study |
| GD006 | Δ*aphA* | EPGS, in-frame deletion in *aphA*, Amp^r^ | This study |
| GD007 | Δ*rpoE*Δ*aphA* | EPGS, in-frame deletion in *rpoE* and *aphA*, Amp^r^ | This study |
| GD008 | Δ*rpoE*Δ*luxR* | EPGS, in-frame deletion in *rpoE* and *luxR*, Amp^r^ | This study |
| GD009 | Δ*degS* | EPGS, in-frame deletion in *degS*, Amp^r^ | This study |
| GD010 | *degS*^+^ | Δ*degS* complemented strain, Amp^r^, Cm^r^ | This study |
| GD011 | Δ*rseA^20-39^* | EPGS, in-frame deletion in *rseA*, Amp^r^ | This study |
| GD012 | *RseA^OE^* | EPGS, overexpression of *rseA*, Amp^r^, Cm^r^ | This study |
| GD013 | pMD19T::P*_rpoE_*-mcherry-P*_rpoH_*-YFP-P*_luxR_*-CFP,pBAD33::*rpoE*-*flag*/DH5α | DH5α, pMD19T carrying P*_rpoE_*-mcherry-P*_rpoH_*-  YFP-P*_luxR_*-CFP, and pBAD33 expressing the *rpoE-flag,*  Amp^r^, Cm^r^ | This study |
| GD014 | pDM8::P*_asp_*/EPGS | EPGS, pDM8 carrying the promoter region of *asp*, Amp^r^, Cm^r^ | This study |
| GD015 | pDM8::P*_asp_*/Δ*rpoE* | Δ*rpoE*, pDM8 carrying the promoter region of *asp*, Amp^r^, Cm^r^ | This study |
| GD016 | pDM8::P*_asp_*/Δ*rpoS* | Δ*rpoS*, pDM8 carrying the promoter region of *asp*, Amp^r^, Cm^r^ | This study |
| GD017 | pDM8::P*_rpoE_*/EPGS | EPGS, pDM8 carrying the promoter region of *rpoE*, Amp^r^, Cm^r^ | This study |
| GD018 | pDM8::P*_rpoS_*/EPGS | EPGS, pDM8 carrying the promoter region of *rpoS*, Amp^r^, Cm^r^ | This study |
| GD019 | pDM8::P*_luxR_*/EPGS | EPGS, pDM8 carrying the promoter region of *luxR*, Amp^r^, Cm^r^ | This study |
| GD020 | pDM8::P*_luxR_*/Δ*luxR* | Δ*luxR*, pDM8 carrying the promoter region of *luxR*, Amp^r^, Cm^r^ | This study |
| GD021 | pDM8::P*_luxR_*/Δ*aphA* | Δ*aphA*, pDM8 carrying the promoter region of *luxR*, Amp^r^, Cm^r^ | This study |
| GD022 | pDM8::P*_luxR_*/Δ*luxO* | Δ*luxO*, pDM8 carrying the promoter region of *luxR*, Amp^r^, Cm^r^ | This study |
| GD023 | pDM8::P*_luxR_*/  Δ*rpoE*Δ*aphA* | Δ*rpoE*Δ*aphA*, pDM8 carrying the promoter region of *luxR*, Amp^r^, Cm^r^ | This study |
| GD024 | pDM8::P*_luxR_*/  Δ*rpoE*Δ*luxR* | Δ*rpoE*Δ*luxR*, pDM8 carrying the promoter region of *luxR*, Amp^r^, Cm^r^ | This study |
| GD025 | pDM8::P*_luxR_493*  /EPGS | EPGS, pDM8 carrying the 493bp of *luxR* promoter, Amp^r^, Cm^r^ | This study |
| GD026 | pDM8::P*_luxR_*ΔLBSI  /EPGS | EPGS, pDM8 carrying the deletion LuxRI box of *luxR* promoter, Amp^r^, Cm^r^ | This study |
| GD027 | pDM8::P*_luxR_*ΔLBSII  /EPGS | EPGS, pDM8 carrying the deletion LuxRII box of *luxR* promoter, Amp^r^, Cm^r^ | This study |
| GD028 | pDM8::P*_luxR_*ΔLBS-I/  II /EPGS | EPGS, pDM8 carrying the deletion LuxRIand LuxRII box of *luxR* promoter, Amp^r^, Cm^r^ | This study |
| GD029 | pDM8::P*_luxR_* ΔRpoEB /EPGS | EPGS, pDM8 carrying the deletion RpoE box of *luxR* promoter, Amp^r^, Cm^r^ | This study |
| GD030 | pDM8::P*_luxR_*ΔABS/  EPGS | EPGS, pDM8 carrying the deletion AphA box of *luxR* promoter, Amp^r^, Cm^r^ | This study |
| GD031 | pET22b::*rpoE*/BL21 | BL21, pET22b carrying the *rpoE* ORF, Amp^r^ | This study |
| GD032 | pET28a::*aphA*/BL21 | BL21, pET28a carrying the *aphA* ORF, Km^r^ | This study |
| RHP003 | pET28a::*luxR*/BL21 | BL21, pET28a carrying the *luxR* ORF, Km^r^ | (Rui et al., 2009) |
| GD033 | pUT18C::*zip*,pKT25::*zip*/BTH101 | BTH101, pUT18C carrying *zip* and pKT25 carrying *zip*,  Amp^r^, Km^r^ | This study |
| GD034 | pUT18C::*rpoE*, pKT25/BTH101 | BTH101, pUT18C carrying *rpoE* ORF and pKT25, Amp^r^, Km^r^ | This study |
| GD035 | pUT18C, pKT25  ::*rseA*/BTH101 | BTH101, pKT25 carrying *rseA* ORF and pUT18C, Amp^r^, Km^r^ | This study |
| GD036 | pUT18C::*rpoE*, pKT  25::*rseA*/BTH101 | BTH101, pUT18C carrying *rpoE* ORF and pKT25 carrying *rseA* ORF, Amp^r^, Km^r^ | This study |
|  | **Plasmids** |  |  |
|  | pDM4 | Suicide vector, *pir* dependent, R6K, *SacBR*, Cm^r^ | (Wang et al., 2002) |
|  | pMMB206 | IncQ lacI^q^ Δ*bla* P_tac-lac_ *lacZa*, Cm^r^ | (Morales et al., 1991) |
|  | pBAD33 | Ara induecd expressing vector, Cm^r^ | (J Beckwith et al.,1995) |
|  | pBAD33-mob | pBAD33 carrying a mob gene, Cm^r^ | This study |
|  | pET22b | Expressing vector, Km^r^ | Novagen |
|  | pET28a | Expressing vector, Km^r^ | Novagen |
|  | pKT 25 | Expressing vector, Km^r^ | Euromedex |
|  | pUT18C | Expressing vector, Amp^r^ | Euromedex |
|  | pKT 25-*zip* | pKT25 carrying the Leucine zipper, Km^r^ | Euromedex |
|  | pUT18C-*zip* | pUT18C carrying the Leucine zipper, Amp^r^ | Euromedex |
|  | pDM4::*rpoE* | pDM4 with *rpoE* fragment deleted 4 to 576 nt, Cm^r^ | This study |
|  | pDM4::*aphA* | pDM4 with *aphA* fragment deleted 4 to 573 nt, Cm^r^ | This study |
|  | pDM4::*rseA* | pDM4 with *rseA* fragment deleted 58 to 117 nt, Cm^r^ | This study |
|  | pDM4::*degS* | pDM4 with *degS* fragment deleted 4 to 1098 nt, Cm^r^ | This study |
|  | pDM4::up*lacZ*down | pDM4 with *lacZ* insert in *glmS*, Cm^r^ | This study |
|  | pBAD33::*rpoE* | pBAD33 derivative *rpoE* expression plasmid, Cm^r^ | This study |
|  | pBAD33::*rpoE-flag* | pBAD33 derivative *rpoE-flag* expression plasmid, Cm^r^ | This study |
|  | pMMB206::*rseA* | pMMB206 derivative *rseA* expression plasmid, Cm^r^ | This study |
|  | pMMB206::*degS* | pMMB206 derivative *degS* expression plasmid, Cm^r^ | This study |
|  | pMD19T ::P*_rpoE_*-mcherry-P*_rpoH_*-YFP-P*_luxR_*-CFP | pMD19T derivative with  P*_rpoE_*-mcherry-P*_rpoH_*-YFP-P*_luxR_*-CFP, Amp^r^ | This study |
|  | pET22b::*rpoE* | pET22b carrying the *rpoE* ORF, Amp^r^ | This study |
|  | pET28a::*aphA* | pET28a carrying the *aphA* ORF, Km^r^ | This study |
|  | pET28a::*luxR* | pET28a carrying the *luxR* ORF, Km^r^ | (Rui et al., 2009) |
|  | pDM8::*asp* | pDM8 carrying the promoter region of *asp*, Cm^r^ | This study |
|  | pDM8::*rpoE* | pDM8 carrying the promoter region of *rpoE*, Cm^r^ | This study |
|  | pDM8::*rpoS* | pDM8 carrying the promoter region of *rpoS*, Cm^r^ | This study |
|  | pDM8::*luxR* | pDM8 carrying the promoter region of *luxR*, Cm^r^ | This study |
|  | pDM8::*luxR493* | pDM8 carrying the 493bp of *luxR* promoter, Cm^r^ | This study |
|  | pDM8::P*_luxR_* ΔLBSI | pDM8 carrying the deletion LuxR1 box of *luxR* promoter, Cm^r^ | This study |
|  | pDM8::P*_luxR_*ΔLBSII | pDM8 carrying the deletion LuxR2 box of *luxR* promoter, Cm^r^ | This study |
|  | pDM8::P*_luxR_*ΔLBS-I/  II | pDM8 carrying the deletion LuxR1 and LuxR2box of *luxR* promoter, Cm^r^ | This study |
|  | pDM8::P*_luxR_* ΔRpoEB | pDM8 carrying the deletion RpoE box of *luxR* promoter, Cm^r^ | This study |
|  | pDM8::P*_luxR_*ΔABS | pDM8 carrying the deletion AphA box of *luxR* promoter, Cm^r^ | This study |
|  | pUT18C::*rpoE* | pUT18C derivative *rpoE* expression plasmid, Amp^r^ | This study |
|  | pKT25::*rseA* | pKT25 derivative *rseA* expression plasmid, Km^r^ | This study |

**References:**

1. Liang WL, Wang SX, Yu FG, Zhang LJ, Qi GM, Liu YQ, Gao SY, Kan B. (2003) Construction and evaluation of a safe, live, oral *Vibrio cholerae* vaccine candidate, IEM108. Infect Immun 71:5498–5504. doi: 10.1128/IAI.71.10.5498-5504 PMID: 14500467

2. Rui H, Liu Q, Wang Q, Ma Y, Liu H, Shi C, et al. (2009) Role of alkaline serine protease, asp, in *Vibrio alginolyticus* virulence and regulation of its expression by LuxO-LuxR regulatory system. J Microbiol Biotechnol 19: 431–438. doi: 10.4014/jmb.0807.404 PMID: 19494689

3. Liu H, Gu D, Cao X, Liu Q, Wang Q, Zhang Y. (2012) Characterization of a new quorum sensing regulator *luxT* and its roles in the extracellular protease production, motility, and virulence in fish pathogen *Vibrio alginolyticus*. Arch Microbiol 194: 439–452. doi: 10.1007/s00203-011-0774-x PMID: 22130678

4. Wang SY, Lauritz J, Jass J, Milton DL. (2002) A ToxR homolog from *Vibrio anguillarum* serotype O1 regulates its own production, bile resistance, and biofilm formation. J Bacteriol 184:1630–1639. doi: 10.1128/JB.184.6.1630-1639.2002 PMID: 11872714

5. Morales VM, Bäckman A, Bagdasarian M. (1991) A series of wide-host range low-copy-number vectors that allow direct screening for recombinants. Gene 97:39–47. doi:10.1016/0378-1119(91) 90007-X PMID: 1847347

6. L M Guzman, D Belin, M J Carson and J Beckwith. (1995) Tight regulation, modulation, and high-level expression by vectors containing the arabinose PBAD promoter. J Bacteriol 177:4121– 4130. PMID: 7608087
