# Supplementary material for: A σE-Mediated Temperature Gauge Controls a Switch from LuxR-Mediated Virulence Gene Expression to Thermal Stress Adaptation in Vibrio alginolyticus
Source: PLoS Pathog. 2016 Jun 2;12(6):e1005645. doi: 10.1371/journal.ppat.1005645 (PMC4890791; doi:10.1371/journal.ppat.1005645)
Supplement: S2 Table — (DOCX) [file ppat.1005645.s010.docx]

**S2 Table. Primers used in this study**

| **Primer name** | Primer sequence (5’ to 3’) | **Target** |
| --- | --- | --- |
| *rseA*1 | GGAAGATCTAAAATCACATTGTTGCTCAAGGGCG | For *rseA* deletion mutant |
| *rseA*2 | CATCACCAATAGCCTTATCGACCAATTCTCCATCC | For *rseA* deletion mutant |
| *rseA*3 | TCGATAAGGCTATTGGTGATGTGATGCGAGGCGA | For *rseA* deletion mutant |
| *rseA*4 | ACATGCATGCACGAGCTCTACCCACTTTTACATAG | For *rseA* deletion mutant |
| *rseA*outF | GGCTGTATCGCATTGCAGTG | For *rseA* deletion mutant |
| *rseA*outR | ACGCAGCACTTGTGTTGCCA | For *rseA* deletion mutant |
| *rseA*comF | GGAAGATCT AGCAAGACCAAGAGAGCCGTGATGC | For *rseA* complement |
| *rseA*comR | ACATGCATGCCAAACTGCGTCAACCAACCTGGCAA | For *rseA* complement |
| *rpoE*1 | GGAAGATCTTGAAAAGTAATAGTTTGTCAATGCC | For *rpoE* deletion mutant |
| *rpoE*2 | TACTTGTACTATCGAGCGGATACTCCTATTTGTTAT | For *rpoE* deletion mutant |
| *rpoE*3 | TATCCGCTCGATAGTACAAGTAATAACTATGGTGAA | For *rpoE* deletion mutant |
| *rpoE*4 | ACATGCATGCTTGAATCGCTACCACCGTACTGCTG | For *rpoE* deletion mutant |
| *rpoE*outF | CGACGTCCGTGTTACGGC | For *rpoE* deletion mutant |
| *rpoE*outR | CAATACAGGTAATTGATCTG | For *rpoE* deletion mutant |
| *rpoE*inF | TGGTGTCAATACTACGTTTTGC | For *rpoE* deletion mutant |
| *rpoE*inR | GTCTACGCCCTTGAGCAACAA | For *rpoE* deletion mutant |
| *rpoE*comF | CCATACCCGTTTTTTTGGGCTAGCGAATTCGGTAACAAA  GTTACCTTGTGTA | For *rpoE* complement |
| *rpoE*comR | GGTCAGCATGGGTACCTTTCTCCTCTTTAAGAAACGCCAT  CTACTGACGGCA | For *rpoE* complement |
| *degS*1 | GGAAGATCTAAAGCGATTAATACTTTCTCAGAGC | For *degS* deletion mutant |
| *degS*2 | CTTGCTTGTCTACATTGAAAAATAACAAAAGGGCA | For *degS* deletion mutant |
| *degS*3 | TTTCAATGTAGACAAGCAAGAAGCATAAAAAATCC | For *degS* deletion mutant |
| *degS*4 | ACATGCATGCCCACAACTGGCGAAAGGTAAAGGGA | For *degS* deletion mutant |
| *degS*out-F | GTCGTACCGGACAGCGCAGC | For *degS* deletion mutant |
| *degS*out-R | GTCTAAAAACCGCAGTGGTA | For *degS* deletion mutant |
| *degS*in-F | TAACTATCATGTGGTTGCTC | For *degS* deletion mutant |
| *degS*in-R | TCCAATATAACCACGAATCA | For *degS* deletion mutant |
| *degS*comF | ACTGGTACCGTCGTACCGGACAGCGCAGCCGA | For *degS* complement |
| *degS*comR | AGCTCTAGAGGTATTCCTAGGTAGTGACGGTC | For *degS* complement |
| *aphA*1 | GGGGTCGACCCGTCCACTCGGTGAAAAACTTCGT | For *aphA* deletion mutant |
| *aphA*2 | CAAAAGGAGTGCTACTTATACTTTCAGCTGCGCTC | For *aphA* deletion mutant |
| *aphA*3 | TATAAGTAGCACTCCTTTTGCTTTGATAAAACGAA | For *aphA* deletion mutant |
| *aphA*4 | ACTTCTAGAGAATTGACGTAGAGATTGTGCCAGG | For *aphA* deletion mutant |
| *aphA*out-F | AGCTTACGTGCAATCCCGTGGATCT | For *aphA* deletion mutant |
| *aphA*out-R | AACGTAAGGTAGATGCAGGTGCA | For *aphA* deletion mutant |
| *aphA*in-F | TTACTTCTGGAAAGCAAGCC | For *aphA* deletion mutant |
| *aphA*in-R | GAGAAACCAGTTTGCGAGAC | For *aphA* deletion mutant |
| P*_rpoE_*-mcherry-1 | GAGCTCAGGTTACCCGCATGCAAGATCTATCTTGTGTATTAT  TTGCACACCATGT | For P*_rpoE_*-mcherry construct |
| P*_rpoE_*-mcherry-2 | CTTGCTCACCATTCGAGCGGATACTCCTATTTGTTAT | For P*_rpoE_*-mcherry construct |
| P*_rpoE_*-mcherry-3 | ATCCGCTCGAATGGTGAGCAAGGGCGAGGAGGA | For P*_rpoE_*-mcherry construct |
| P*_rpoE_*-mcherry-4 | CTGCTCGTTCATTTACTTGTACAGCTCGTCCATGCCG | For P*_rpoE_*-mcherry construct |
| P*_rpoE_*-mcherry-5 | GTACAAGTAAATGAACGAGCAGCTGACCGATCAAG | For P*_rpoE_*-mcherry construct |
| P*_rpoE_*-mcherry-6 | CCCTCGAGTACGCGTCACTAGTGGGGCCCTACTGAACACCA  CCCGTTGCAATTCT | For P*_rpoE_*-mcherry construct |
| P*_luxR_*-CFP-1 | GGTCAGCATGGGTACCTTTCTCCTCTTTAATAAGCCTAC  CAACACAAGATCTT | For P*_luxR_*-CFP construct |
| P*_luxR_*-CFP-2 | TGCTCACCATATCCATTTTCCTTGCCATTTGAG | For P*_luxR_*-CFP construct |
| P*_luxR_*-CFP-3 | AGGAAAATGGATATGGTGAGCAAGGGCGAGGAGCTGT | For P*_luxR_*-CFP construct |
| P*_luxR_*-CFP-4 | TTGAGTCCATTTACTTGTACAGCTCGTCCATGC | For P*_luxR_*-CFP construct |
| P*_luxR_*-CFP-5 | CTGTACAAGTAAATGGACTCAATTGCAAAGAGACC | For P*_luxR_*-CFP construct |
| P*_luxR_*-CFP-6 | CCATACCCGTTTTTTTGGGCTAGCGAATTCTTTGTACCA  GCAACTGGTTAGTA | For P*_luxR_*-CFP construct |
| P*_rpoH_*-YFP-1 | GAGCTCAGGTTACCCGCATGCAAGATCTATACCATGAAACT  GATCGGTGCGAC | For P*_rpoH_*-YFP construct |
| P*_rpoH_*-YFP-2 | CTTGCTCACCATTCAATTCCTCATCAATCTCTGAT | For P*_rpoH_*-YFP construct |
| P*_rpoH_*-YFP-3 | GAGGAATTGAATGGTGAGCAAGGGCGAGGAGCT | For P*_rpoH_*-YFP construct |
| P*_rpoH_*-YFP-4 | CTTTTGTCATTTACTTGTACAGCTCGTCCATGC | For P*_rpoH_*-YFP construct |
| P*_rpoH_*-YFP-5 | CTGTACAAGTAAATGACAAAAGAAGCGTATCCGAT | For P*_rpoH_*-YFP construct |
| P*_rpoH_*-YFP-6 | CCCTCGAGTACGCGTCACTAGTGGGGCCCTGTCGCAATTTTC  ACGATACGCCA | For P*_rpoH_*-YFP construct |
| Pmd19TYFP-F | ATAGATCTTGCATGCGGGTAACCTGAGCTCTATTTCACACCG  CATATGGTGCAC | For P*_rpoH_*-YFP construct |
| Pmd19TYFP-R | AGGGCCCCACTAGTGACGCGTACTCGAGGGCCGCACAGATG  CGTAAGGAGAAA | For P*_rpoH_*-YFP construct |
| pMD19T-1upF | GTGTCGGGGCTGGCTTAACTAT | For P*_rpoH_*-YFP construct |
| pMD19T-1upR | CCGCACCGATCGCCCTTCCCAA | For P*_rpoH_*-YFP construct |
| pMD9T-cfp-F | CCATACCCGTTTTTTTGGGCTAGCGAATTCTGAGCGCAACGC  AATTAATGTGAGT | For P*_luxR_*-CFP construct |
| pMD9T-cfp-R | GGTCAGCATGGGTACCTTTCTCCTCTTTAACTGCCCGCTTTCC  AGTCGGGAAACC | For P*_luxR_*-CFP construct |
| pMD19T-2doF | TCCACACAACATACGAGCCGGA | For P*_luxR_*-CFP construct |
| pMD19T-2doR | GCGGAAGAGCGCCCAATACGCA | For P*_luxR_*-CFP construct |
| *rpoE*pET22b-F | ATCGGATCCGATGAACGAGCAGCTGACCGATC | Protein cloning |
| *rpoE*pET22b-R | ATATGTCGACGCGTTGCAAAAGAGGTCTGATT | Protein cloning |
| *aphA*pET28a-F | ATCGGATCCATGTTTACAAGTTTATTGACCATTT | Protein cloning |
| *aphA*pET28a-R | ATATGTCGACACCAATCACTTCAAGTTCAGTT | Protein cloning |
| *asp*transcript-F | ATCCCGGGGTAAATGCTCAAGACAGTCAACAAC | P*_asp_*-*lacZ* |
| *asp*transcript-R | ATCCCGGGTTGCCCTATTCCTTATTTGAGC | P*_asp_*-*lacZ* |
| *rpoE*transcript-F | ATTCCCGGGGGTAACAAAGTTACCTTGTGTA | P*_rpoE_*-*lacZ* |
| *rpoE*transcript-R | ATTCCCGGGCATTCGAGCGGATACTCCTAT | P*_rpoE_*-*lacZ* |
| *rpoS*transcript-F | ATTCCCGGGGGACAGCGTGGTCAGCCTATCGTTT | P*_rpoS_*-*lacZ* |
| *rpoS*transcript-R | ATTCCCGGGTAACGCCTCCCCCTGGCGAGTTAGC | P*_rpoS_*-*lacZ* |
| *luxR*transcript-F | ATACCCGGGACTTTACCGATGTATGGTAAGTGGC | P*_luxR_*-*lacZ* |
| *luxR*transcript-R | ATACCCGGG ATCCATTTTCCTTGCCATTTGAGTT | P*_luxR_*-*lacZ* |
| *rseA*bacth-F | ATCGGATCCCATGGCTGACAAAGAAAAACTTTCA | Protein cloning |
| *rseA*bacth-R | ATCGAATTCTCATTCAATATCCGGATTCAGATTT | Protein cloning |
| *rpoE*bacth-F | ATCGGATCCCATGAACGAGCAGCTGACCGATCAA | Protein cloning |
| *rpoE*bacth-R | ATCGAATTCTTAGCGTTGCAAAAGAGGTCTGATT | Protein cloning |
| *luxR*EMSAF | AGTCGCACGCAAACGATCACC | EMSA |
| *luxR*EMSAR | ATCCATTTTCCTTGCCATTTG | EMSA |
| *rpoH*EMSA-F | TTGTACGATAGCCAGTTCC | EMSA |
| *rpoH*EMSA-R | CTCTGATCTGGAGCATTACAT | EMSA |
| *rpoE*EMSA –F | AAGATGCTCGCTTGTCAAACT | EMSA |
| *rpoE*EMSA –R | CATTCGAGCGGATACTCCTAT | EMSA |
| VpaF | CCACGCAACAAGCCAACCAAA | EMSA |
| VpaR | CAAAAGGATGTGACTTTCAACCC | EMSA |
| VcoF | CTGCCATAAAGACAAATGAGCCA | EMSA |
| VcoR | CTACGCGCTTAAAATCATCTGGC | EMSA |
| VspF | TCCGCCATAAAGACAAACGATC | EMSA |
| VspR | GAGCAGTGCTAGACTTACGCAC | EMSA |
| P*_luxR_*-493F | AGTCCCGGGAGTCAGTTCGATAGCACGTGCCAA | LacZ assays |
| P*_luxR_*-493R | AGTCCCGGGATCCATTTTCCTTGCCATTTGAGT | LacZ assays |
| P*_luxR_*1-1 | GATACGAACGTCACGTGAGCTTT | LacZ assays |
| P*_luxR_*1-2 | GTAGAACAATTAACTTTATAAAACTAAGACCTTC | LacZ assays |
| P*_luxR_*1-3 | TTTATAAAGTTAATTGTTCTACAGATGTTGACCT | LacZ assays |
| P*_luxR_*1-4 | CTTCGTCACGTGTTGAAGCACTC | LacZ assays |
| P*_luxR_*2-2 | GTAACCCCCTGCTTAAAGCAGTGATGAGTGTAA | LacZ assays |
| P*_luxR_*2-3 | CTGCTTTAAGCAGGGGGTTACCCCCAATATCAAC | LacZ assays |
| P*_luxR_*12-2 | ATGAGTGTAATGGTGCATATGCACAAGGTCAAC | LacZ assays |
| P*_luxR_*12-3 | GCATATGCACCATTACACTCATCACTGCTTTAAGC | LacZ assays |
| P*_luxR_*EB-2 | CTTAAAGCAGTGTAGAACAATTTATATACAGT | LacZ assays |
| P*_luxR_*EB-3 | TTGTTCTACACTGCTTTAAGCAACTATTAAAA | LacZ assays |
| P*_luxR_*AB-2 | TTGAAAGTCGCGTAAGATTAGGTGATCGTTTGCG | LacZ assays |
| P*_luxR_*AB-3 | CCTAATCTTACTTGAAAGTCGCATCCTGTTGCCA | LacZ assays |
| P*_luxR_-*chip-F | GTTCTACAGATGTTGACCTTGTGC | chip |
| P*_luxR_-*chip-R | GGTCTCTTTGCAATTGAGTCCAT | chip |
| P*_rpoE_*-chipF | GACTAAAGTCAGGCAGCGGT | chip |
| P*_rpoE_*-chipR | CGTAGTATTGACACCACTGCTTA | chip |
| P*_rpoH_*-chipF | CCTGATTGGTCTAAGTTGGGATG | chip |
| P*_rpoH_*-chipR | AATCCAAGTAATAAGACCGCCAT | chip |
| GyrB-chip-F | GCTGCGCTAACACGTACATTG | chip |
| GyrB-chip-R | TGAGAACTTAGGATCAGGCACTTT | chip |
| Z-Up-F | AGTACGCGTCACTAGTGGGGCCCTTCTAGAAGAAATTGA  AGCACTAGCAACGG | For EPGS *lacZ*^+^ |
| Z-Up-R | GACTTCAGATCATGCCGCTTGGATTTTTGCTGTTA | For EPGS *lacZ*^+^ |
| *lacZ*-F | AATCCAAGCGGCATGATCTGAAGTCATCCGTAATCA | For EPGS *lacZ*^+^ |
| *lacZ*-R | CACATACTCTCGCATTATTGTGGGGATGACGCTT | For EPGS *lacZ*^+^ |
| Z-Down-F | TCCCCACAATAATGCGAGAGTATGTGCCGCTTTTT | For EPGS *lacZ*^+^ |
| Z-Down-R | TAACAATTTGTGGAATTCCCGGGAGAGCTCTTGAAGAGTGT  CATCATCTCTA | For EPGS *lacZ*^+^ |
| P*_rpoE_*vitro-F | TCCATGCTCGACCCAGCACAATC | In vitro transcription |
| P*_rpoE_*vitro-R | GGTACTTTGTTACCAACAGGTTG | In vitro transcription |
| P*_luxR_*vitro-F | AGTCGCACGCAAACGATCACCT | In vitro transcription |
| P*_luxR_*vitro-R | CGGCGCGCAAATACTTCCAATG | In vitro transcription |
| LuxRfor | CCCAATATCAACTCAAATG | In vitro transcription |
| LuxRrew | GCAAATACTTCCAATGCGA | In vitro transcription |
| RpoEfor | CCATATCTGAGAAGAGTAGG | In vitro transcription |
| RpoErew | TGTTACCAACAGGTTGAATG | In vitro transcription |
| RACE-adapter | GCGCGAATTCCTGTAGA | 5'-RACE |
| RACE-adapter2 | GCGCGAATTCCTGTAGAACGAAC | 5'-RACE |
| *luxR*-RACE-nested | GACCGATGCCGCGGCGCGCAAATAC | 5'-RACE |
| *luxR*-RACE | CTTGGCTGACAAGCTCGATCATCGC | 5'-RACE |
| RNA-Linker | AUAUGCGCGAAUUCCUGUAGAACGAACACUAGAAGAAA | 5'-RACE |
| q*asp*-F | GGTAACAACAATGCCCACGG | For qRT-PCR |
| q*asp*-R | GCCCAGATGAGTAACCCCAAC | For qRT-PCR |
| q*luxR*-F | CACGCGAAGACTTGGTGGAT | For qRT-PCR |
| q*luxR*-R | TGACAGTCTTGGCTGACAAGCT | For qRT-PCR |
| q*mviN*-F | GCTCCCGATAGGGCAACAA | For qRT-PCR |
| q*mviN*-R | CGGAGTGGTGACTGCACTGT | For qRT-PCR |
| q*pep*F | CAAGTGCTTCCGACACTGAA | For qRT-PCR |
| q*pep*R | ACCCGTACGGTTTACCTGTG | For qRT-PCR |
| 16S RNA-F | AAAGCACTTTCAGTCGTGAGGAA | For qRT-PCR |
| 16S RNA-R | TGCGCTTTACGCCCAGTAAT | For qRT-PCR |
